# Evaluation of the cyto- and genotoxicity of two types of cellulose nanomaterials using human intestinal cells and in vitro digestion simulation

**DOI:** 10.1007/s00204-024-03911-2

**Published:** 2024-12-24

**Authors:** Nádia Vital, Maria Cardoso, Michel Kranendonk, Maria João Silva, Henriqueta Louro

**Affiliations:** 1 Department of Human Genetics, National Institute of Health Dr. Ricardo Jorge, Avenida Padre Cruz, 1649-016 Lisbon, Portugal; 2 NOVA Medical School, Universidade NOVA de Lisboa, 1169-056 Lisbon, Portugal; 3 Centre for Toxicogenomics and Human Health (ToxOmics), NOVA Medical School, Universidade NOVA de Lisboa, 1169-056 Lisbon, Portugal

**Keywords:** Cellulose nanomaterials, In vitro digestion, New approach methodology, Intestinal epithelial cells, Micronucleus, Reactive oxygen species, Comet

## Abstract

Emerging cellulose nanomaterials (CNMs) may have commercial impacts in multiple sectors, being their application particularly explored in the food sector. Thus, their potential adverse effects in the gastrointestinal tract should be evaluated before marketing. This work aimed to assess the safety of two CNMs (CNF–TEMPO and CMF–ENZ) through the investigation of their cytotoxicity, genotoxicity (comet and micronucleus assays), and capacity to induce reactive oxygen species in human intestinal cells, and their mutagenic effect using the *Hprt* gene mutation assay. Each toxicity endpoint was analysed after cells exposure to a concentration-range of each CNM or to its digested product, obtained by the application of a standardized static in vitro digestion method. The results showed an absence of cytotoxic effects in intestinal cells, up to the highest concentration tested (200 µg/mL or 25 µg/mL, for non-digested and digested CNMs, respectively). Of note, the cytotoxicity of the digestion control limited the top concentration of digested samples (25 µg/mL) for subsequent assays. Application of a battery of in vitro assays showed that CNF–TEMPO and CMF–ENZ do not induce gene mutations or aneugenic/clastogenic effects. However, due to the observed DNA damage induction, a genotoxic potential cannot be excluded, even though in vitro digestion seems to attenuate the effect. The lowest digested CNF–TEMPO concentration induced chromosomal damage in Caco-2 cells, leading to an equivocal outcome. Ongoing research on epigenotoxic effects of these CNMs samples may strengthen the lines of evidence on their safety when ingested, paving the way for their innovative application in the food industry.

**Supplementary Information:**

The online version contains supplementary material available at 10.1007/s00204-024-03911-2.

## Introduction

The interest in using cellulose nanomaterials (CNMs) or nanocelluloses in various applications across many fields is growing rapidly. Among these, several food-related and food-contact material applications are anticipated, including the reduction and/or substitution of currently used petroleum-based materials for food packaging (Vital et al. 2022a). Moreover, application of CNMs in the biomedical and pharmaceutical fields, for example in drug delivery systems, wound healing and tissue repair, medical implants, vascular grafts, and bone tissue engineering, is under investigation (Trache et al. 2020). Most applications of CNMs in food technology and biomedicine are still in the early R&D stages but are expected to reach commercialization soon. An important reason for the wide interest in CNMs is the easy accessibility of the feedstock material, cellulose—an abundant biodegradable organic polymer—which can be isolated from different sources, but is found mainly in wood or plants (Moon et al. 2011; Trache et al. 2020; Vital et al. 2022a). The group of CNMs includes but is not limited to cellulose nanofibrils (CNFs), with dimensions typically of 3–100 nm in cross section and up to 100 μm in length (ISO 2023). These are mainly obtained by breaking cellulose fibres into delaminated individual nanofibrils via high-energy mechanical shearing methods, preceded by chemical or enzymatic hydrolysis pre-treatments to increase the nanofibrillation efficiency and reduce production costs (Moon et al. 2011; Abdul Khalil et al. 2014). Enzymatic treatments are carried out by a special group of enzymes, cellulases, which catalyse the breakdown of cellulose polymer into smaller polymer branches or cellobiose and glucose (Lourenço et al. 2019; Ribeiro et al. 2019). Common chemical pre-treatments include catalytic oxidation with 2,2,6,6-tetramethylpiperidine-1-oxyl radicals (TEMPO), converting the primary hydroxyl groups on the C6 position of cellulose to carboxylic groups (Saito and Isogai 2004; Saito et al. 2007; Gamelas et al. 2015; Lourenço et al. 2017; Levanič et al. 2020; Vital et al. 2022a). The different production methods can originate CNFs with distinct physicochemical characteristics such as length, width, aspect ratio, degree of polymerization (i.e., the number of glucose units), surface chemistry, and crystallinity (Foster et al. 2018; Trache et al. 2020). By manipulating these characteristics one can modulate the thermal, mechanical, optical, and rheological properties, tunable for the type of application.

In the USA, the Food and Drug Administration (FDA) designates celluloses as “generally regarded as safe” (GRAS) for their intended uses in food and food contact materials, and permits their safe use, provided it meets specific criteria, according to the CFR—Code of Federal Regulations Title 21 (FDA n.d.). Several types of celluloses and their derivatives are authorized in food, as food additives (e.g., thickener, emulsifier, binder, stabilizer, and gelling agent), under the European Regulation (EC) No 231/2012, and in food packaging, under the European Regulation (EC) No 10/2011. Have been considered safe for use as food additives in food and animal feed, based on their low toxicity, absence of genotoxic properties, and, if any, their negligible absorption through the gastrointestinal tract (GIT) (EFSA Panel on Food Additives and Nutrient Sources added to Food et al. 2018). However, it has been recognized that the reduced size and the corresponding increased and modified surface area of nanomaterials (NMs), might alter their physiological fate and behaviour, and may raise concerns about adverse health effects compared to micron-sized or larger sizes (Kohl et al. 2020; Siivola et al. 2022; Schoonjans et al. 2023).

Human exposure to CNMs is likely to occur through different routes. Inhalation is regarded as the main route of exposure to CNMs in humans, particularly in occupational settings, and therefore most studies, both in vitro and in vivo, are focused on the respiratory tract (Ventura et al. 2020). Additionally, CNMs have raised some concerns due to their fibre-like morphology resembling the persistent high-aspect-ratio nanomaterials (HARNs), such as some multi-walled carbon nanotubes (MWCNT-7), which have been associated with pulmonary adverse biological effects given the fibre pathogenicity paradigm (Endes et al. 2016; IARC 2017). Yet, considering the above-mentioned applications, as well as the potential contamination of water and landfills and indirect ingestion of inhaled CNMs, the oral route is an important route of exposure. A more thorough assessment of CNMs safety in oral exposure is still missing, especially considering the primary site of contact of CNMs, the GIT, upon their ingestion (Vital et al. 2022a; Brand et al. 2022). Although an increasing number of studies have addressed toxicity of CNMs in the last few years (Ventura et al. 2020; Brand et al. 2022; Vital et al. 2022a), the potential genotoxic effect of CNMs in the GIT has not yet been fully clarified. Different types of CNMs showed non-significant direct toxic effects *in* vitro assays (Deloid et al. 2019; Salari et al. 2019; Pradhan et al. 2020; Zhang et al. 2021; Vincentini et al. 2023), except exposure to (i) concentrations above 2000 µg/mL (Tibolla et al. 2018, 2019) or, (ii) to carboxymethylated CNFs, a type of functionalization of CNFs (Lopes et al. 2020). Regarding CNFs treated with a static in vitro simulated digestion process, no relevant cytotoxic effect or oxidative stress has been reported when evaluated with intestinal co-culture cellular models (Deloid et al. 2019; Salari et al. 2019; Pradhan et al. 2020; Patel et al. 2021). Likewise, in Caco-2 cells, CNFs did not induce ROS generation, apoptotic marker active Caspase-3, marker NF-kB nor IL-8 cytokine secretion, using High Content Analysis (HCA) (Vincentini et al. 2023). Moreover, all studied CNMs, except one of the cellulose nanocrystals (CNC), did not induce increased phosphorylation of histone variant H2AX producing γH2AX and phosphorylation of ATM at S1981, both DNA damage markers (Vincentini et al. 2023). To our knowledge, no in vitro study addressed regulatory accepted genotoxicity endpoints (micronuclei and/or chromosomal aberrations) applying intestinal cells after exposure to CNFs. The few i*n vivo* toxicity studies investigating CNMs effects upon ingestion, reported an overall absence of toxic effects. This was even the case when testing high doses of CNFs, based on different parameters from clinical pathology, anatomic pathology, histopathology, hematology, serum chemistry, and urinalysis (Ong et al. 2020; Andrade et al. 2015; Deloid et al. 2018, 2019; Zhang et al. 2021; Chen et al. 2020). One study showed altered rodents' microbiome, expression of epithelial cell junction genes, and increased production of cytokines by CNF (Khare et al. 2020). Another study described CNF induced disturbance in glucose homeostasis and decreased intestinal absorption (Chen et al. 2020). These mentioned studies were not specifically designed to evaluate genotoxicity in rodents’ intestinal cells. However, differences in the physiology and uptake of the NMs in the GIT between humans and rodents have been pointed out to hamper an adequate risk assessment of NMs in those models (Sohal et al. 2018).

The use of simulated human digestion may provide a valuable early stage tool for hazard characterization of NMs when used as a complementary approach to in vitro toxicological testing in human intestinal cellular models, as an alternative to animal models. Such human in vitro digestion models mimic the conditions of the GIT, including mouth, stomach, and intestine. Each of these compartments represents different conditions, such as pH, temperature, bile salts, ionic strength, digestion time and digestive enzymatic activities, thus simulating the different in vivo environments (Minekus et al. 2014; Brodkorb et al. 2019). These human digestion approaches emulate the physiological process of ingested NMs, moving along these different compartments, which may modify the physicochemical properties, bioavailability, and thus the toxicological profile of NMs. The standardized INFOGEST protocol is considered by EFSA as a key approach in the toxicity evaluation of NMs to which humans may be orally exposed (EFSA Scientific Committee et al. 2021). This approach has been coupled to the general set of genotoxicity tests used for the safety assessment of ingested NMs, as recently reported for titanium dioxide nanomaterials (Bettencourt et al. 2020; Vieira et al. 2022). More recently, the adaptation of the INFOGEST protocol has been described, to mitigate the toxic impact of digestion products for testing nanomaterials, allowing its application in the biological assays (Vital et al. 2024).

The present study describes the in vitro investigation of intestinal cyto- and genotoxicity of two CNMs, namely a CNF produced by catalytic oxidation with TEMPO radical (CNF–TEMPO) and a CMF produced by enzymatic hydrolysis (CMF–ENZ). This evaluation was performed using also a new approach methodology for simulating human digestion in vitro prior to genotoxicity tests in the intestinal cell models Caco-2 and HT29-MTX-E12, in adding an important physiological process to this in vitro model for oral nanotoxicology. For the evaluation of gene mutation, the pulmonary V79 cells were used as a recommended cell line for addressing such endpoint.

## Materials and methods

### CNM’s characteristics

CNF–TEMPO and CMF–ENZ were obtained from industrial bleached *Eucalyptus globulus* kraft pulp (BEKP), refined in a PFI Beater, constituted by 80–85 wt% cellulose, 14–19 wt% xylan, 0.3 wt% lignin and 0.4 wt% extractives. Details on CNF–TEMPO and CMF–ENZ production are described elsewhere (Pinto et al. 2022; Ventura et al. 2023). Briefly, to obtain the CNF–TEMPO, the refined fibres were subjected to a TEMPO-mediated oxidation, for 2h, by adding 0.016 g of radical TEMPO, 0.1 g of NaBr and 5 mM of NaOCl per gram of fibres. To synthesize the CMF–ENZ, an enzymatic hydrolysis treatment with 10% endocellulase, 10% exocellulase, and 5% hemicellulose, was applied to the refined fibres at 50 ºC for 2 h, at a dosage of 300 g/ton of fibres. After the chemical and enzymatic treatments, CNF–TEMPO and CMF–ENZ were washed with distilled water and mechanically homogenised in a high-pressure homogenizer (GEA Niro Soavi, model Panther NS3006 L, GEA Group Aktiengesellschaft, Düsseldorf, Germany) with 2 passages (at 500 bar and at 1000 bar) (Pinto et al. 2022; Ventura et al. 2023).

The characterization of the primary properties of both CNMs, such as the fibrillation yield, carboxyl content (C_COOH_), degree of polymerization (DP), and intrinsic viscosity (η), was described previously by our group (Pinto et al. 2022). The carboxyl content was higher in CNF–TEMPO (1332 μmol/g) than in CMF–ENZ (143 μmol/g), while presenting a lower degree of polymerization (309) and intrinsic viscosity (130 ml/g); CMF–ENZ showed a degree of polymerization of 1591 and an intrinsic viscosity of 618 ml/g (Pinto et al. 2022). The morphology and estimated diameter, when dispersed in phosphate buffered saline (PBS), were also previously analysed by Transmission Electron Microscope (TEM) using the negative staining technique (Pinto et al. 2022). The mean diameters were 10.7 ± 1.9 nm and 29.7 ± 7.3 nm, for CNF–TEMPO and CMF–ENZ), respectively.

To complement data on secondary properties of CNMs, electrophoretic light scattering using a Zetasizer Nanoseries Nano Z (Malvern Instruments, Malvern, UK) was used to measure the surface charge by determining the Zeta potential of the CNMs samples dispersed in PBS and in complete cell culture medium (at a concentration of 14.3 µg/mL). Zeta potential was also determined in the simulated digestion end products (digested samples). All measurements were performed in triplicate and the results are shown as the mean ± standard deviation (SD).

### CNM’s preparation and concentration range selection

Stock suspensions of CNMs, at a concentration of 1.5 mg/mL PBS were prepared by dispersing with magnetic stirring for 30 min. The stock dispersions were used immediately, either for the digestion protocol (resulting in the digested samples, DIG, as described in Section "In vitro digestion protocol and reagents") or directly for the biological assays (corresponding to the undigested samples) described in further sections.

The choice of top dose of 200 µg/mL for undigested samples was limited by the dispersibility of the CNMs, as observed under microscopy. To define the concentration range of CNMs to be used after digestion (DIG samples), a preliminary cytotoxicity testing was performed with the digestion product without the presence of CNMs, using the MTT assay, considering that the simulated digestion fluids used for simulated digestion are known to be cytotoxic (Vital et al. 2024). Based on the results, a concentration up to 25 µg/mL was selected for testing the CNMs in the subsequent cytotoxicity and genotoxicity assays.

Considering the reported effects of the digestion product per se (Vital et al. 2024), five digestion controls were used in subsequent assays to allow the comparison between digested CNMs-treated cells and their respective “digestion control” (DIG Control, C1–C5). The percentage of digestion product present in the culture medium corresponding to the CNMs concentrations are the following: 3.1 μg/mL–1.7% (C1); 6.3 μg/mL–3.4% (C2); 14.3 μg/mL–7.6% (C3); 25 μg/mL–13.3% (C4); 50 μg/mL–26.7% (C5).

The MTT assay was also used for preliminary evaluation of the dose range to use in V79 cells in the Hprt (hypoxanthine phosphoribosyltransferase) gene mutation assay (data not shown). For assays in V79 cells, the digested negative controls are the percentage of digestion product present in the culture medium corresponding to the CNMs concentrations as following: 1.6 μg/mL–0.85% (C1), 3.1 μg/mL–1.7% (C2); 6.3 μg/mL–3.4% (C3); 14.3 μg/mL–7.6% (C4); 25 μg/mL–13.3% (C5); 50 μg/mL–26.7% (C6). We tested concentrations ranging from 1.6 to 200 μg/mL (undigested samples) and from 1.6 to 50 μg/mL (digested samples). There was a significant 21–25% decrease in viability for undigested CNF–TEMPO at 100 and 200 μg/mL (*p* = 0.029 and 0.0168, Student’s *t* test). Also, a 20 or 48% significant decrease in viability was detected when testing the digestion product without CNMs at concentrations equivalent to 3.1 (C2; *p* = 0.006) or 6.3 μg/mL (C3; *p* < 0.0001), without CNMs, and > 90% cytotoxicity was reached at 14.3, 25 and 50 μg/mL (C4, C5 and C6). For these reasons, the range of concentrations selected for the gene mutation assay was from 1.6 to 100 μg/mL (undigested samples) and 1.6 to 6.3 μg/mL (digested samples), since higher concentrations up to 200 μg/mL (undigested samples) and 50 μg/mL (digested samples) presented higher cytotoxicity that could interfere with the genotoxicity assessment.

Before the exposure of cells for the genotoxicity assays, undigested and DIG CNMs samples were diluted in complete Dulbecco’s modified Eagle’s medium (DMEM, Thermo Fisher, Waltham, MA, USA) to obtain the desired concentrations, ranging from 3.1 to 200 µg/mL (undigested CNMs) or 3.1–25 µg/mL (digested samples).

### In vitro* digestion protocol and reagents*

The study used the standardized static INFOGEST 2.0 in vitro digestion protocol (Minekus et al. 2014; Brodkorb et al. 2019) with the modification of the bile salt concentration, as previously reported (Vital et al. 2024). The protocol includes three sequential phases of digestion—oral, gastric and intestinal. At each phase, a digestive fluid with a specific composition is added, i.e., simulated salivary fluid (SSF; pH 7), simulated gastric fluid (SGF; pH 3), and simulated intestinal fluid (SIF; pH 7), respectively. Detailed description of the content of the salts/electrolytes concentration of the simulated fluids were reported previously (Vital et al. 2024). The following reagents were used to prepare the simulated digestion fluids: (NH_4_)_2_CO_3_ (Sigma-Aldrich, St. Louis, MO, USA), CaCl_2_**·**2H_2_O, KCl, MgCl_2_**·**6H_2_O, NaHCO_3_, NaCl, NaOH (Merck, Darmstadt, Germany), HCl and KH_2_PO_4_ (J. T. Baker, Center Valley, PA, USA). Additionally, depending on the digestive phase, different enzymes and other constituents were sequentially added to an initial volume of 1 mL of PBS (control), CNF–TEMPO or CMF–ENZ, as follows: (i) 1 mL of SSF (1X) with 75 U/mL α-amylase, 1.5 mM of CaCl_2_.2H_2_O and Milli-Q water, mixed in a mechanical shaker for 2 min at 37 °C (oral phase); (ii) 2 mL of SGF (1X) with 2000 U/mL pepsin, 0.15 mM of CaCl_2_.2H_2_O and Milli-Q water, mixed for 120 min at 37 °C (gastric phase); (iii) 4 mL of SIF(1X) with 100 U/mL pancreatin, 4 mM bovine bile, 0.6 mM of CaCl_2_.2H_2_O and Milli-Q water, mixed for 120 min at 37 °C (intestinal phase). At the end of the intestinal phase, the enzymatic activity was stopped with Pefabloc® SC (5 mM). Pepsin, pancreatin, α-Amylase, bovine bile, and Pefabloc®SC, were purchased from Sigma-Aldrich® (St. Louis, MO, USA). The final solution obtained at the end of the in vitro digestion protocol includes the sum of all the components added during the sequential phases, thereafter, denominated as “digestion product”.

### Intestinal cell culture conditions and reagents

Two human intestinal cell lines—Caco-2 and HT29-MTX-E12 cell lines (European Collection of Authenticated Cell Cultures, ECACC, Salisbury, UK), were selected as experimental models for in vitro studies. The human colon adenocarcinoma cell line Caco-2 was chosen due to its source tissue (human colon), the characteristics resembling human enterocytes, and its common use *in *in vitro studies. The human colorectal adenocarcinoma HT29-MTX-E12 cells (mucous-secreting), a subclone of HT29 cells differentiated into mature goblet cells using methotrexate, was selected as an alternative intestinal model, with the ability to produce a mucous layer, as informed by the cell line description catalogue. Both cell lines were routinely maintained under standard cell culture conditions (37 °C, 5% of CO_2_) in Dulbecco's Modified Eagle Medium (DMEM) with 4.5 g/L glucose, supplemented with 10% fetal bovine serum (FBS), 1% penicillin/streptomycin (10,000 U/mL), 1% Amphotericin B (0.25 mg/mL), and 2.5% HEPES Buffer (all reagents were obtained from Thermo Fisher, Waltham, MA, USA). Cells were regularly checked for the absence of mycoplasma contamination by PCR amplification. Cells were detached from the flasks with trypsin–EDTA (0.05%, Invitrogen, Carlsbad, California, USA), and seeded in appropriate densities to perform the assays.

Chinese hamster lung fibroblast cells (V79, ECACC, Salisbury, UK) were used for the gene mutation assay. Cells were grown in low glucose DMEM GlutaMAX™ Supplement, supplemented with 1% Amphotericin B (Fungizone; 0.25 mg/mL), 1% Penicillin/Streptomycin (10,000 U penicillin/10 μg streptomycin), 2.5% HEPES Buffer and 10% FBS (all reagents were obtained from Thermo Fisher).

### Cytotoxicity assessment

Cytotoxicity of CNMs was evaluated in Caco-2 and HT29-MTX-E12 cells using complementary in vitro approaches, spanning different endpoints, such as metabolic activity (MTT reduction) and cell proliferation (colony formation). Additionally, the cytostasis/cytotoxicity was also evaluated through the cytokinesis block proliferation index (CBPI) and Replication Index (RI), using the CBMN assay (as described in the next section).

#### MTT assay

The 3-(4,5-dimethylthiazol-2-yl)−2–5-diphenyltetrazolium bromide (MTT, Sigma-Aldrich®, St. Louis, MO, USA) assay was performed on both CMF–ENZ and CNF–TEMPO, in Caco-2 and HT29-MTX-E12 cells, as previously described (Vital et al. 2024). Briefly, 2 × 10^4^ cells per well were cultured in 96-well plates, for 24 h (37 °C, 5% CO_2_). Cells were then exposed, for 24 h, to the above-mentioned concentrations of each undigested and DIG CNMs. Sodium dodecyl sulphate (SDS; 0.01%, for 1 h, Sigma-Aldrich®, St. Louis, MO, USA) was used as a positive control. Untreated (cell culture medium only) or vehicle treated (digested PBS, DIG Control, C1–C4) cells were used as negative controls. At the end of exposure, cells were washed with PBS and incubated for 3 h with MTT (0.5 mg/mL). Afterward, the MTT solution was removed, and cells were incubated for 30 min with dimethyl sulfoxide (DMSO; Sigma-Aldrich®, St. Louis, MO, USA), at room temperature, under constant shaking and protected from light. The absorbance was measured at 570 nm (reference filter: 690 nm), using a Multiscan Ascent spectrophotometer (Thermo LabSystems, Waltham, MA, USA). The relative viability (%) of the treated cells was defined as the ratio of absorbance compared to control (untreated) cells (100% viability). At least three independent experiments were performed per exposure condition, each using six replicate cultures.

#### Clonogenic assay

The clonogenic assay was based on published procedures (Louro et al. 2019; Rundén-Pran et al. 2022), with some modifications. Briefly, before treatment, a very low density of Caco-2 (200 cells per well) or HT29-MTX-E12 (100 cells per well) cells were cultured in a 6-well plate for 24 h, at 37 °C and 5% CO_2_. Then, cells were exposed to the above-mentioned concentrations of each undigested and DIG CNMs, and cultured to allow for colony formation, for 9 days (HT29-MTX-E12) or 15 days (Caco-2), at 37 °C and 5% CO_2_. For each experiment, untreated cells (cell culture medium only) and positive controls (0.05 μg/mL and 0.025 μg/mL mitomycin C, Sigma, for Caco-2 and HT29-MTX-E12, respectively, for 24h) were included. After the exposure period, the wells were washed, fixed in absolute methanol (Sigma) for 15 min, and stained with 10% Giemsa (Merck, Darmstadt, Germany) in phosphate buffer (pH 6.8). The number of colonies formed was counted and the cloning efficiency (CE) was determined using the following equation (Herzog et al. 2007): CE = 100 × (no. colonies in negative control/no. of plated cells). The cell viability or surviving fraction (SF) was calculated for each concentration as SF = no. colonies formed after exposure/ (no. of plated cells × CE/100). The cytotoxicity was determined as the decrease in the SF relative to the negative control, from four independent experiments each with three replicates per exposure condition.

### Genotoxicity evaluation

#### Cytokinesis-blocked micronucleus assay (CBMN) assay

The cytokinesis-block micronucleus (CBMN) assay was performed according to the OECD 487 international guideline, modified to overcome the potential interference of NMs with the assay (Louro et al. 2016; OECD 2023). Briefly, HT29-MTX-E12 cells and Caco-2 cells were seeded and incubated for 24 h (37 °C, 5% CO_2_), after which cells were exposed to undigested and DIG CNMs. Cytochalasin-B (4.5 µg/mL, Sigma-Aldrich, St. Louis, MO, USA) was added 24 h after exposure; the cells were then incubated for an additional 28 h (total exposure time of 52 h). For each experiment, negative controls—untreated or vehicle treated (digested PBS, DIG Control, C1–C5) cells and positive controls (0.3 μg/mL mitomycin C, Sigma-Aldrich, St. Louis, MO, USA) were included. At the end of the treatment, the cells were submitted to a hypotonic shock with KCL (0.1 M) and fixed immediately with cold methanol/acetic acid solution (3:1 v/v), followed by centrifugation. The fixation step, followed by centrifugation, was repeated one more time, and then the pellet was spread onto microscope slides, stained with 4% Giemsa (Merck, Darmstadt, Germany), and air-dried at room temperature. The slides were coded and blind-scored under a bright field microscope (Axioskop 2 Plus, Zeiss, Germany), for the presence of micronucleated binucleated cells (MNBC) using the criteria described elsewhere (Fenech 2007). 2000 binucleated cells (BC) from two independent cultures were scored per treatment condition, equally divided among the cultures. The frequency of micronucleated binucleated cells per 1000 cells (MNBC/1000 BC) was determined. The proportion of mono-, bi-, or multinucleated cells was determined in a total of 1000 cells per treatment, and the cytokinesis-blocked proliferation index (CBPI), as well as the replication Index (RI), was calculated using OECD guideline (OECD 2023), as follows:

$$\text{CBPI}=\frac{[\text{Number of mononucleate cells}]+2\times [\text{Number of binucleate cells}]+3\times [\text{Number of multinucleate cells}]}{[\text{Total number of cells}]}.$$

#### Comet assay

Both the conventional and the formamidopyrimidine DNA glycosylase (FPG)-modified versions of the comet assay were performed. The cells were plated at a density of 7 × 10^4^ cells per well in 12-well plates and incubated for 24 h, at 37°C and 5% CO_2_, before exposure. After 3 h and 24 h exposure to each undigested and DIG CNMs samples, cells were harvested, and the alkaline comet assay was performed as described elsewhere (Vieira et al. 2022). Briefly, at the end of exposure, the cell suspension was centrifuged, the pellets were resuspended and mixed with 0.8% low melting point agarose (Sigma-Aldrich) and then spread onto microscope slides pre-coated with 1% normal melting point agarose. After an overnight lysis step (NaCl 2.5 M, Na_2_EDTA.2H_2_O 100 mM, Tris–HCl 10 mM; pH 10; 10% DMSO and 1% Triton-X100), the slides were washed with the FPG enzyme reaction buffer (F buffer: HEPES 40 mM, KCl 100 mM, acid EDTA 0.5 mM, BSA 0.2 mg/ml; pH 8) and incubated either with F buffer or with FPG enzyme (New England Biolabs, Ipswich, MA, USA), for 30 min, in a humidified chamber at 37 °C. The slides were then immersed into cold electrophoresis buffer (NaOH 0.3 M, Na_2_EDTA.2H_2_O 1 mM; pH 13) for 30 min, followed by 25 min electrophoresis at 0.8 V/cm. Finally, slides were neutralized, dried overnight, and stained with ethidium bromide (0.125 μg/μL) before analysis under a fluorescence microscope (Leica Dm500, Germany) using the comet Assay IV image analysis system (Perceptive Instruments, UK). The median of the percentage of DNA in the tail (% DNA in tail) was chosen as a measure of DNA damage. The results represent the mean ± Standard Deviation (SD) of the median of at least two independent experiments, each with two replicates per treatment condition, in which 100 cells were scored per treatment condition, in 2 gels (50 nucleoids per gel). The Net FPG-sensitive sites were calculated as the difference of the percentage of DNA in the tail between the enzyme-incubated and reaction-buffer-incubated samples. Ethyl methanesulfonate (EMS, 5 mM; Sigma-Aldrich, St. Louis, MO, USA) was used as a positive control, for 1 h exposure. Untreated or vehicle treated (digested PBS, DIG Control, C1–C4) cells were used as negative controls.

#### *Mammalian *in vitro* Hprt gene mutation test*

This assay was performed according to the OECD 476 international guidelines for the in vitro mammalian cell gene mutation tests using the *Hprt gene* (OECD 2016), as described elsewhere (Vital et al. 2022b), with some modifications. Prior to the assay V79 cells were first grown in Hypoxanthine–Aminopterin–Thymidin (HAT) medium (complete medium with 100 µM hypoxanthine, 0.4 µM aminopterin and 16 µM thymidine) for 5 days to eliminate preexisting *Hprt* gene mutants. Cells were seeded 24 h before exposure at a density of 0.5 × 10^6^ cells per flask and then exposed for 24 h to 1.6, 3.1, 6.3, 14.3 and 100 μg/mL of non-digested CNMs and 1.6, 3.1, 6.3 μg/mL of digested CNMs and incubated at 37 ºC with 5% CO_2_. For each experiment, negative controls—untreated or vehicle treated (digested PBS, DIG Control, C1–C3) cells and positive control (EMS, 3 mg/mL, 30 min; Sigma-Aldrich) were included. At the end of exposure, the cells were then seeded in 100 mm petri dishes and maintained in exponential growth for 7 days to allow phenotypic expression of *Hprt* mutants: the medium was removed, flasks were washed, trypsinized and re-suspended in 5 mL medium (on days 3 and 5) and incubated at 37 ºC with 5% CO_2_. Samples were taken for analysis of mutant frequencies at 7 and 9 days (1st and 2nd harvesting) after treatment, by subculturing in 100 mm diameter Petri dishes (3 × 10^5^ cells/Petri dish, 6 dishes per sample concentration) and grown in selective mem α medium (Gibco) containing 6-thioguanine (Sigma) at 5 µg/mL for 8 days to allow colony formation. Then, mutant (6-thioguanine-resistant) colonies were stained with 1% methylene blue (Sigma) and counted manually (only colonies with at least 50 cells were considered).

The frequency of surviving cells was assessed using the plating efficiency (PE) assay after exposure (PE_0,_) and for each of the two harvests (8 and 10 days, PE_1_ and PE_2_, respectively). Treated and untreated cells were seeded in 6-well plates at 50 cells per well (6 wells per condition) and incubated for 7 days at 37ºC. The colonies formed were stained with 1% methylene blue (Sigma-Aldrich) for 20 min and counted manually.

Mutant Frequency (MF) and Platting Efficiency (PE) were calculated according to the following equation:

$$\text{Mutant Frequency }({10}^{-6})=\frac{\text{Number of mutant colonies}}{\text{Number of surviving inoculated cells}}\times 100$$

$$\text{PE}(\text{\%})=\frac{\text{Number of colonies counted}}{\text{Number of inoculated cells}}\times 100$$

Within the experiment, cytotoxicity after exposure (RPE, relative plating efficiency) were calculated based on the PE_0_ according to the equation:

$$\text{RPE}(\text{\%})= \frac{\text{PE of exposure cultures}}{\text{PE of unexposed cultures}}\times 100$$

### Intracellular reactive oxygen species (ROS) measurement

The intracellular ROS levels were determined with the probe 2′,7′-Dichlorofluorescin diacetate (DCFDA, Sigma-Aldrich, Saint Louis, MO, USA), as previously described (Vieira et al. 2022). Briefly, Caco-2 and HT29-MTX-E12 cells were seeded separately at equal density (2 × 10^4^ cells per well, 100 μL per well) in black 96-well microplates with clear bottom, and incubated for 24 h, at 37 °C and 5% CO_2_. Cells were then incubated with 20 μM of DCFDA, for 30 min, in the dark at 37 °C. The probe solution was removed, and cells were incubated, for 3 and 24 h, with undigested and DIG CNMs samples, at 37 °C and 5% CO_2._ Hydrogen peroxide solution (250 µM, 1 h) was used as a positive control for the induction of ROS. Untreated or vehicle treated (digested PBS, DIG Control, C1–C4) cells were used as negative controls. The fluorescent 2,7-dichlorofluorescein (DCF) levels were determined at excitation 485 nm and emission 535 nm wavelengths using SpectraMax ID3 (Molecular Devices, San Jose, CA, USA). Relative ROS level is expressed as a fold-change of relative fluorescence units (RFU) of exposed cells compared to relative fluorescence units (RFU) in control cells, from three experiments.

### Statistical analysis and interpretation of results

The statistical analysis was performed using IBM SPSS Statistics 26 (Armonk, NY, USA) or Prism software (5, GraphPad, San Diego, CA, USA). The two-tailed Fisher’s exact test was applied to analyse the results of the frequency of micronucleated binucleated cells between exposed and non-exposed cells. Provided that the data followed a normal distribution, statistical comparisons of MTT, CBPI, ROS and comet assays data between treated and control cells, after exposure to non-digested samples, were performed through one-way analysis of variance (ANOVA). When necessary, the ANOVA was followed by Dunnett´s post hoc tests to analyse for differences between the different concentrations of CNMs and the negative control. The two-tailed Student's *t* test was used to compare the differences between the results of MTT, CBPI, ROS and comet assays data between treated and control cells, after exposure to digested samples comparatively to the respective digested control; for comparisons of samples with and without FPG treatment; or with and without digestion. Non-parametric tests such as the Kruskal–Wallis or the Mann–Whitney *U* test were applied in all other cases. Data are expressed as the average of 2–4 independent experiments ± SD. Differences with a *p* value lower than 0.05 were considered statistically significant.

According to the OECD recommendations for general chemicals, a response is considered a clear positive in a specific genotoxicity test if it meets all the criteria below in at least one experimental condition (OECD 2017): (i) at least one of the data points exhibits a statistically significant increase compared to the concurrent negative control; (ii) the increase is concentration- or dose-related at least at one sampling time when evaluated with an appropriate trend test; (iii) the result is outside the distribution of the historical negative control data (e.g., Poisson-based 95% control limits).

According to OECD, a test chemical is considered clearly negative if, in all experimental conditions examined, none of the above OECD criteria for a positive result are met and a lack of genotoxicity is recognized. If the response is neither clearly negative nor clearly positive, is concluded to be equivocal (interpreted as equally likely to be positive or negative).

To evaluate the biological relevance of the effect, the confidence intervals of the means for the concurrent controls and the treated cultures were also evaluated and compared with the historical control data (e.g., 95% control limits), as recommended (OECD 2017). Therefore, only significant differences falling outside the 95% confidence limits of concurrent negative control and negative historical control data intervals were considered relevant and presented as statistically significant.

For results not clearly negative or positive according to the above-mentioned criteria, additional judgment specific for NMs, based on Nanogenotox Final Report (NanoGenoTox 2023), was used as follows: + + + , POSITIVE—significant dose-dependent increase and ≥ 2 significant doses; + + , POSITIVE—significant dose-dependent increase and high dose significant; + , POSITIVE—no significant dose-dependent increase and ≥ 2 significant doses; ( +), EQUIVOCAL—no significant dose-dependent increase, 1 significant dose; -, NEGATIVE.

## Results

### CNMs surface charge

The surface charge of the two (digested and undigested) CNMs samples dispersed in PBS (stock dispersion), or cell culture medium is shown in Table 1. All dispersions presented negative surface charge. Regarding CNF–TEMPO, both PBS and cell culture medium dispersions presented negative zeta potentials that were not significantly affected by digestion. On the contrary, the surface charge of CMF–ENZ (in PBS or medium) decreased significantly after in vitro digestion.

**Table 1:** Determination of surface charge (Zeta potential, mV) of the CNMs dispersions in PBS and cell culture medium (14.3 µg/mL), with and without digestion simulation

CNMs	Undigested		Digested	
	PBS	Cell culture medium	PBS	Cell culture medium
CMF–ENZ	– 10.1 ± 0.7	– 6.0 ± 0.4	– 20.4 ± 1.2*	– 21.8 ± 1.3*
CNF TEMPO	– 21.3 ± 1.2	– 11.6 ± 2.7	– 21.9 ± 0.9	– 16.4 ± 1.7

Results are presented as mean ± SD (*n* = 3)

*Significantly different from undigested counterpart sample (*P*=0.0042, Student’s *t* test)

### Cytotoxicity evaluation

Cytotoxicity of CNMs was evaluated in Caco-2 and HT29-MTX-E12 cells using complementary in vitro approaches, spanning different processes, such as metabolic activity (MTT reduction) and cell proliferation (colony formation; MTT). Additionally, the cytotoxicity was also evaluated through the cytokinesis block proliferation index (CBPI) and Replication Index (RI), using the CBMN assay.

**Fig. 1:**
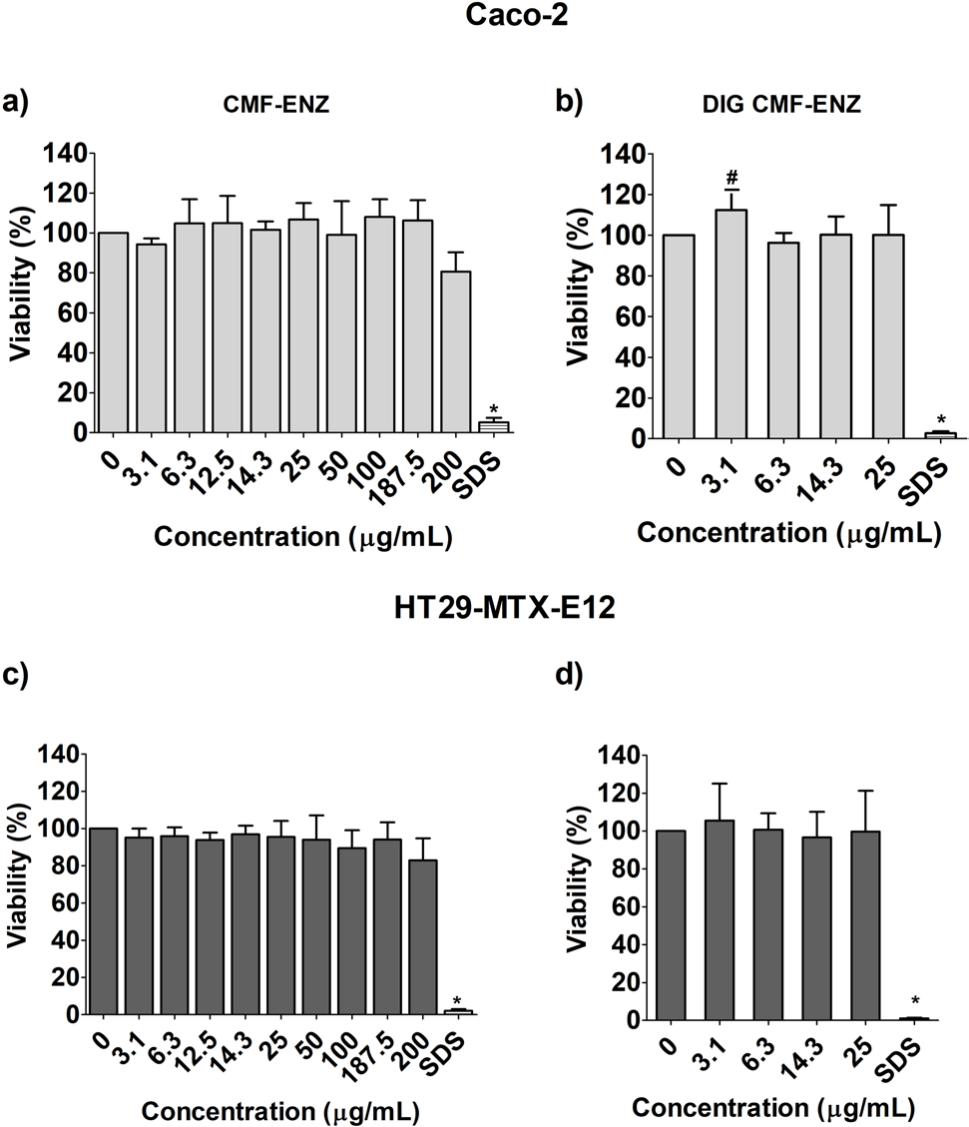
Cell viability (MTT assay) of Caco-2 and HT29-MTX-E12 cells after exposure to undigested and digested CMF–ENZ. Results are presented as mean cell viability ± Standard deviation (*N* = 3), relative to the respective negative or digestion controls (all control reads were set to correspond to 100% viability). **#**—significantly different from undigested CNMs. Positive control SDS (sodium dodecyl sulfate: 0.01%, for 1 h)

**Fig. 2:**
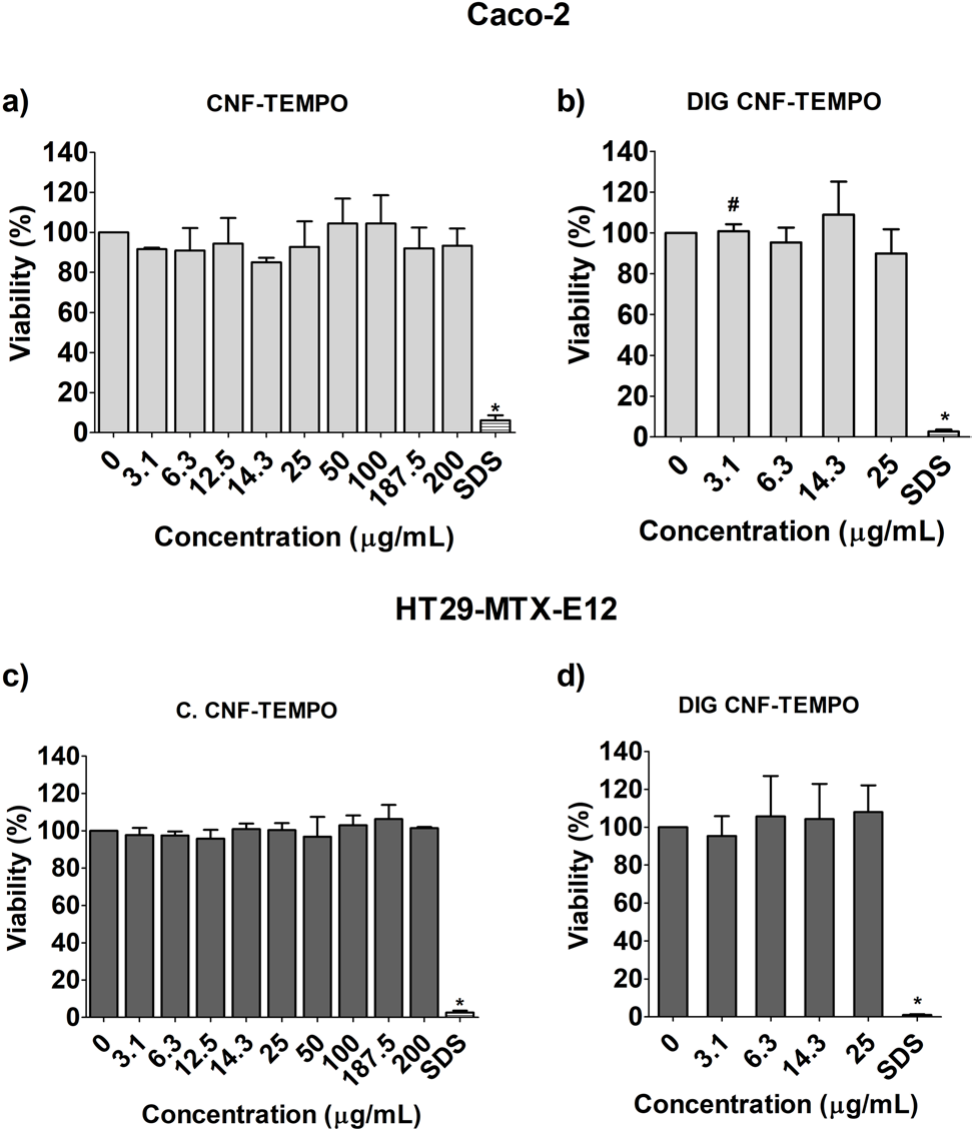
Cell viability (MTT assay) of Caco-2 and HT29-MTX-E12 cells after exposure to undigested and digested CNF–TEMPO. Results are presented as mean cell viability ± Standard deviation (*N* = 3), relative to the respective negative or digestion controls (all control reads were set to correspond to 100% viability). **#**—significantly different from undigested CNMs. Positive control SDS (sodium dodecyl sulfate: 0.01%, for 1 h)

The results of the cytotoxicity assessment of both CNMs applying the MTT assay are presented in Figs. 1a–d and 2a–d. None of the tested CNMs induced cytotoxicity, after 24 h of exposure up to 200 µg/mL, both in Caco-2 cells (Figs. 1a, 2a) or in HT29-MTX-E12 cells (Figs. 1c, 2c). Likewise, no significant cytotoxicity was observed after in vitro digestion of the two CNMs, compared to the respective digestion controls, after exposure to up to 25 µg/mL, in Caco-2 Cells and HT29-MTX-E12 cells. A small non-significant decrease of viability was observed with the none-digested samples after exposure to 3.1 μg/mL of both CNMs. A significant, but less than 1.2-fold increase in viability, was observed after Caco-2 cells’ exposure to 3.1 μg/mL of digested CMF–ENZ (Fig. 1b; *p* = 0.0305) and CNF–TEMPO (Fig. 2b; *p* = 0.0062) when compared to the respective undigested counterparts, possibly related with the small viability decrease observed with the none-digested samples. The positive control (SDS 0.1%) was cytotoxic in both cell lines as expected, yielding a relative viability that ranged from 0.6 to 9.1% in all experiments.

The clonogenic assay was performed following cells’ exposure to each sample for 9 days (HT29-MTX-E12) or 15 days (Caco-2) (Fig. S1). No cytotoxicity was observed in Caco-2 cells for any of the evaluated CNMs. However, with HT29-MTX-E12 cells, a mild decrease in clone formation efficiency (< 20%) was observed after exposure to both CNMs compared to the negative control (CMF–ENZ: *p* < 0.0001; CNF–TEMPO: *p* = 0.0453; one-way ANOVA). Based on the ISO standard 10,993–5, this decrease can be considered of non-significance as the observed relative toxicity was lower than 30% (ISO 2009). When testing the digested CNMs, no colony formation was observed irrespective of the concentration, due to the cytotoxicity of the digestion product, with the long-term exposures used.

The results of the cytotoxicity assessment through the determination of the cytokinesis block proliferation index (CBPI) and Replication Index (RI), applying the CBMN assay, are presented in Figs. S2 and S3. Concerning the digestion controls (digestion product without CNMs), cells exposure to the highest percentage of the digestion control (C5, equivalent sample contents of 50 μg/mL of CNMs), for 52 h, led to cytotoxicity in both cell lines (Fig. S2c, f). Based on the above-described toxic effect of the digestion product, the concentration range for the MN assay with digested CNMs was selected between 3.1 and 14.3 μg/mL with the respective digestion controls (C1–C3), to stay within the maximum of 55% for recommended by OECD TG 487 (OECD 2017).

Undigested and digested samples of CMF–ENZ (Fig. S2) and CNF–TEMPO (Fig. S3) did not show any significant alteration in CBPI or RI of both Caco-2 or HT29-MTX-E12 cells’, as compared to their respective negative controls.

### DNA damage: comet assay

**Fig. 3:**
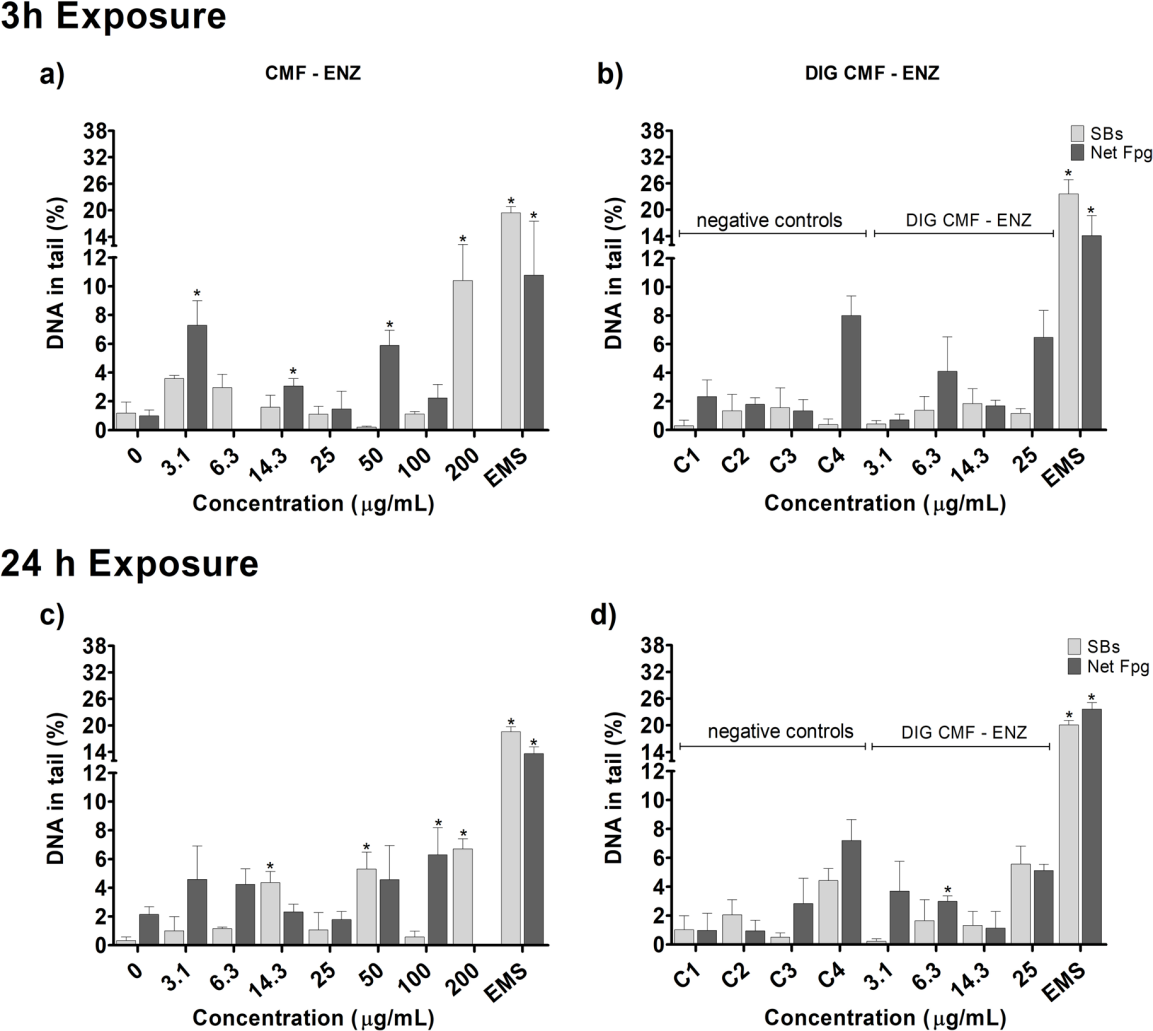
DNA damage (comet assay) with Caco-2 cells after exposure 3 h and 24 h to undigested and digested CMF–ENZ. 0—negative control; C1–C4—DIG 0 controls. Results are presented as mean ± Standard deviation (N = 2). *—significantly different from the respective negative control. SBs—DNA strand breaks. Net FPG—DNA oxidation lesions (Net Fpg-sensitive sites). Positive control EMS (Ethyl methanesulfonate: 5 mM, 1 h)

**Fig. 4:**
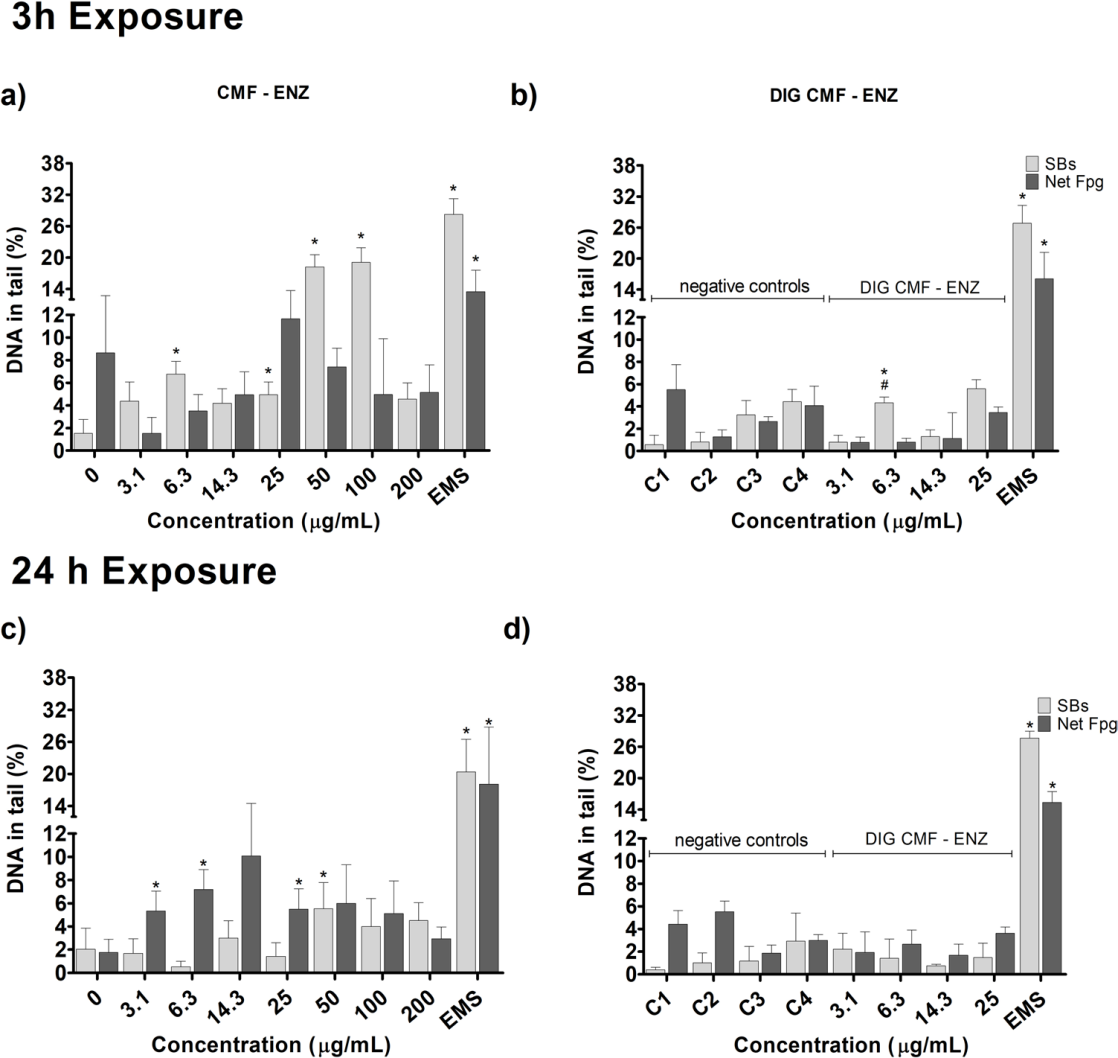
DNA damage (comet assay) with HT29-MTX-E12 cells after exposure 3 h and 24 h to undigested and digested CMF-ENZ. 0—negative control; C1–C4—DIG 0 controls. Results are presented as mean ± Standard deviation (N = 2). *—Significantly different from the respective negative control. #—Significantly different from the undigested sample. SBs—DNA strand breaks. Net FPG—DNA oxidation lesions (Net Fpg-sensitive sites). Positive control EMS (Ethyl methanesulfonate: 5 mM, 1 h)

**Fig. 5:**
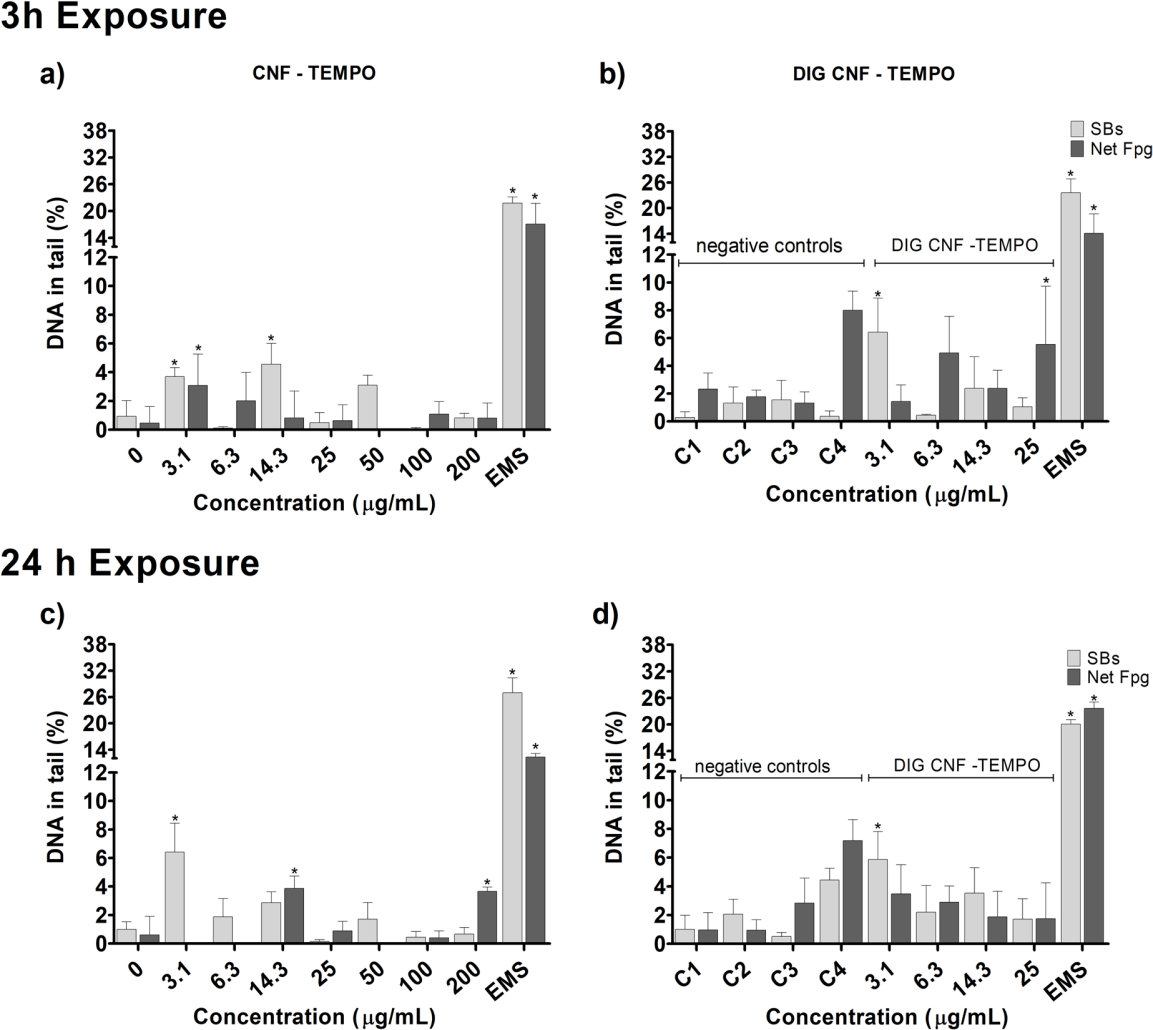
DNA damage (comet assay) with Caco-2 cells, after 3 h and 24 h exposure to undigested and digested CNF-TEMPO. 0—negative control; C1–C4—DIG 0 controls. Results are presented as mean ± Standard deviation (N = 2). *—Significantly different from the respective negative control. SBs—DNA strand breaks. Net FPG—DNA oxidation lesions (Net Fpg-sensitive sites). Positive control EMS (Ethyl methanesulfonate: 5 mM, 1 h)

**Fig. 6:**
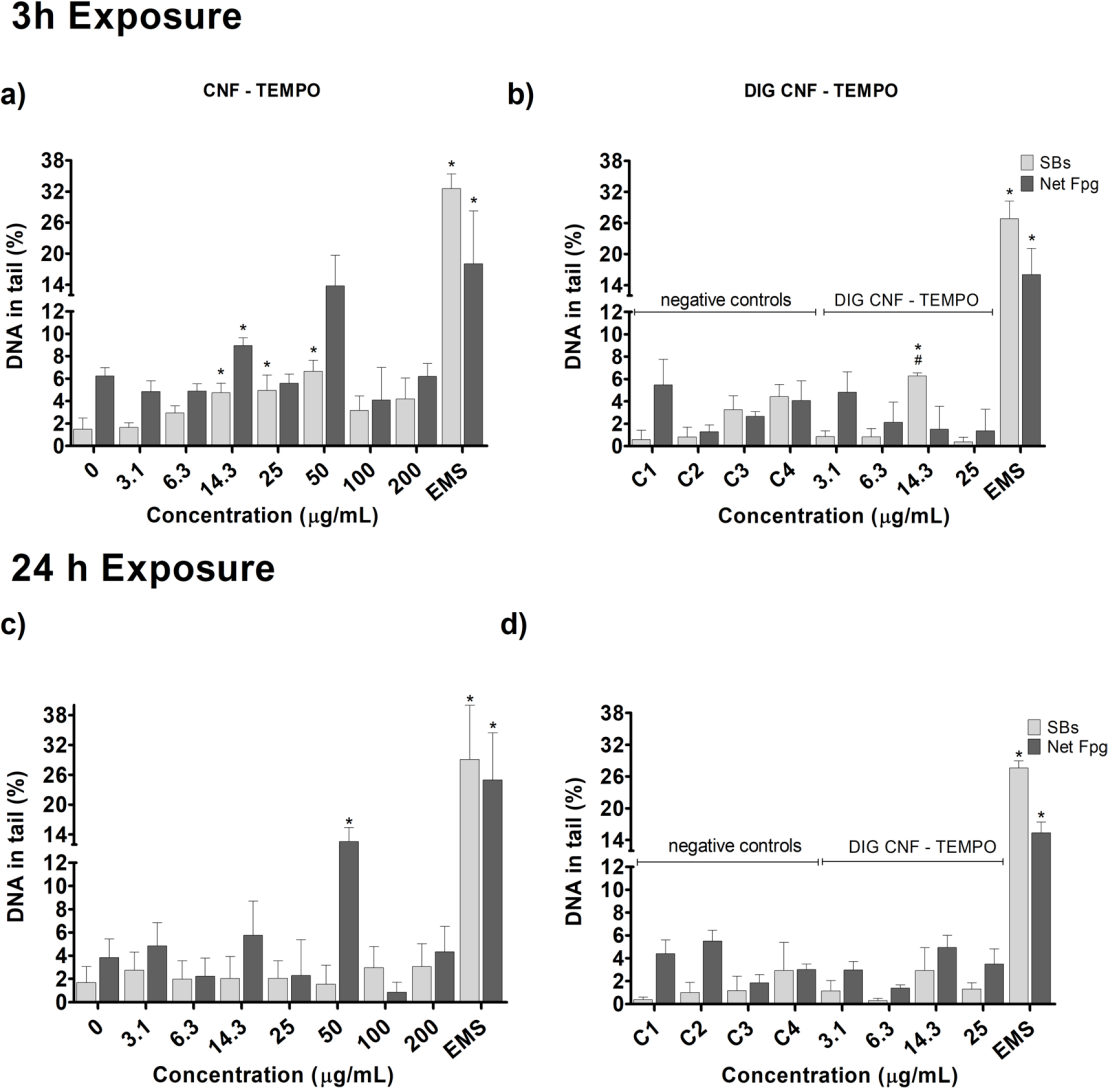
DNA damage (comet assay) with HT29-MTX-E12, after 3 h and 24 h exposure to undigested and digested CNF–TEMPO. 0—negative control; C1–C4—DIG 0 controls. Results are presented as mean ± Standard deviation (*N* = 2). *—Significantly different from the respective negative control. #—Significantly different from the undigested sample. SBs—DNA strand breaks. Net FPG—DNA oxidation lesions (Net Fpg-sensitive sites). Positive control EMS (Ethyl methanesulfonate: 5 mM, 1 h)

Evaluation of DNA damage, as assessed by the percentage of DNA in tail, by the two types of CNMs in the Caco-2 and HT29-MTX-E12 cell models was performed by the conventional and FPG-modified comet assays (Net FPG-sensitive sites), after 3 h and 24 h exposure. Results are presented in Figs. 3, 4, 5 and 6.

CMF–ENZ caused a significant increase in DNA damage, in Caco-2 cells, after a 3h exposure to the maximum tested concentration (200 μg/mL) (Fig. 3a; *p* = 0.009). Exposure to the digested CMF–ENZ did not produce DNA damage, compared to the respective control, up to 25 μg/mL (Fig. 3b). The FPG-comet assay revealed a significant increase in DNA oxidation lesions, after 3 h incubation with 3.1, 14.3 and 50 μg/mL of undigested CMF–ENZ (Fig. 3a), but no increase was observed with the digested CMF–ENZ at corresponding concentrations (Fig. 3b). Regarding the 24 h exposures, 14.3 μg/mL, 50 μg/mL, and 200 μg/mL of CMF–ENZ significantly increased the level of DNA damage (Fig. 3c; *p* = 0.006, *p* = 0.002 and *p* = 0.0005, respectively). A significant increase in the level of DNA oxidation lesions was observed only at 100 μg/mL (Fig. 3c). In turn, Caco-2 cells exposure to 6.3 μg/mL of digested CMF–ENZ for 24 h resulted in DNA oxidation lesions (Fig. 3d; *p* = 0.0026).

In HT29-MTX-E12 cells, the same 3 h incubation revealed significant differences in the level of DNA damage at 6.3, 25, 50 and 100 μg/mL of CMF–ENZ, compared to the negative control (Fig. 4a; *p* < 0.00001). The digested CMF–ENZ increased the level of DNA damage in cells exposed to 6.3 μg/mL for 3h, compared with the respective digestion control (Fig. 4b; *p* = 0.033). No effect was observed in the level of DNA oxidation lesions at 3h exposure (Fig. 4a,b). After 24 h exposure, a DNA damaging effect (SBs) was observed (Fig. 4c), but only with 50 μg/mL of CMF–ENZ. However, increased oxidation DNA damage (Net Fpg) was already observed with 3.1, 6.3 and 25 μg/mL of CMF–ENZ (Fig. 4c;* p* = 0.042, *p* = 0.003 and *p* = 0.039, respectively). For digested CMF–ENZ, no DNA damage effects were observed, comparatively with the respective control (Fig. 4d).

Regarding CNF–TEMPO, 3 h of exposure of Caco-2 cells to 3.1 μg/mL and 14.3 μg/mL induced very low, but significant levels of DNA damage, with less than 5% DNA in tail (Fig. 5a). This digested CNF–TEMPO 3 h exposure increased significantly the level of DNA damage at the lowest concentration tested (*p* = 0.0286), comparatively to the respective digestion control (Fig. 5b). With the same incubation time, increased oxidation DNA lesion levels were seen after exposure to CNF–TEMPO at 3.1 μg/mL, but not for DIG-TEMPO. A 24 h exposure to CNF–TEMPO (Fig. 5c) caused a significant increase in the % of DNA in the tail at the lowest concentrations tested (3.1 μg/mL; *p* = 0.0001), an effect also observed after exposure to the same concentrations of the digested sample (Fig. 5d; *p* = 0.004), comparatively to the respective digestion control. For the same time point, increased oxidation DNA lesion levels were seen after exposure to CNF–TEMPO at 14.3 μg/mL and 200 μg/mL compared to its control (*p* = 0.002 and *p* = 0.004, respectively).

Using the HT29-MTX-E12 cell model, CNF–TEMPO induced DNA damage after 3h exposure to 14.3, 25 and 50 μg/mL, comparatively to the respective negative control (Fig. 6a; *p* < 0.0001). For the same incubation time, the digested sample induced a low increase in the percentage of DNA in the tail only at the concentration of 14.3 μg/ mL, compared to the respective digestion controls (Fig. 6b; *p* = 0.0187) and compared to the undigested sample (Fig. 6a vs b, *p* = 0.0085). Induction of oxidation DNA damage was observed after cells treatment with 14.3 μg/mL (*p* < 0.0001), but only for the undigested sample (Fig. 6a). No DNA damaging effects were observed for 24 h exposures to CNF–TEMPO (Fig. 6c) and its digested counterpart (Fig. 6d). Increased levels of oxidation lesions were detected only after cells exposure to 50 μg/mL of undigested CNF–TEMPO (Fig. 6C; *p* = 0.0027).

Noteworthy, in Caco-2 cells, there was DNA damage effect of the digestion product itself, hampering the determination of the real effect of the digested CNMs after 24 h exposure to C4 (corresponding to 25 μg/mL). Induction of oxidation DNA damage was observed for 3 h treatments with digestion control C4. Positive controls induced a significant increase in DNA damage and oxidation lesions, confirming the sensitivity of the assay to detect DNA single- and double-strand breaks.

### Chromosomal damage: micronucleus assay

**Fig. 7:**
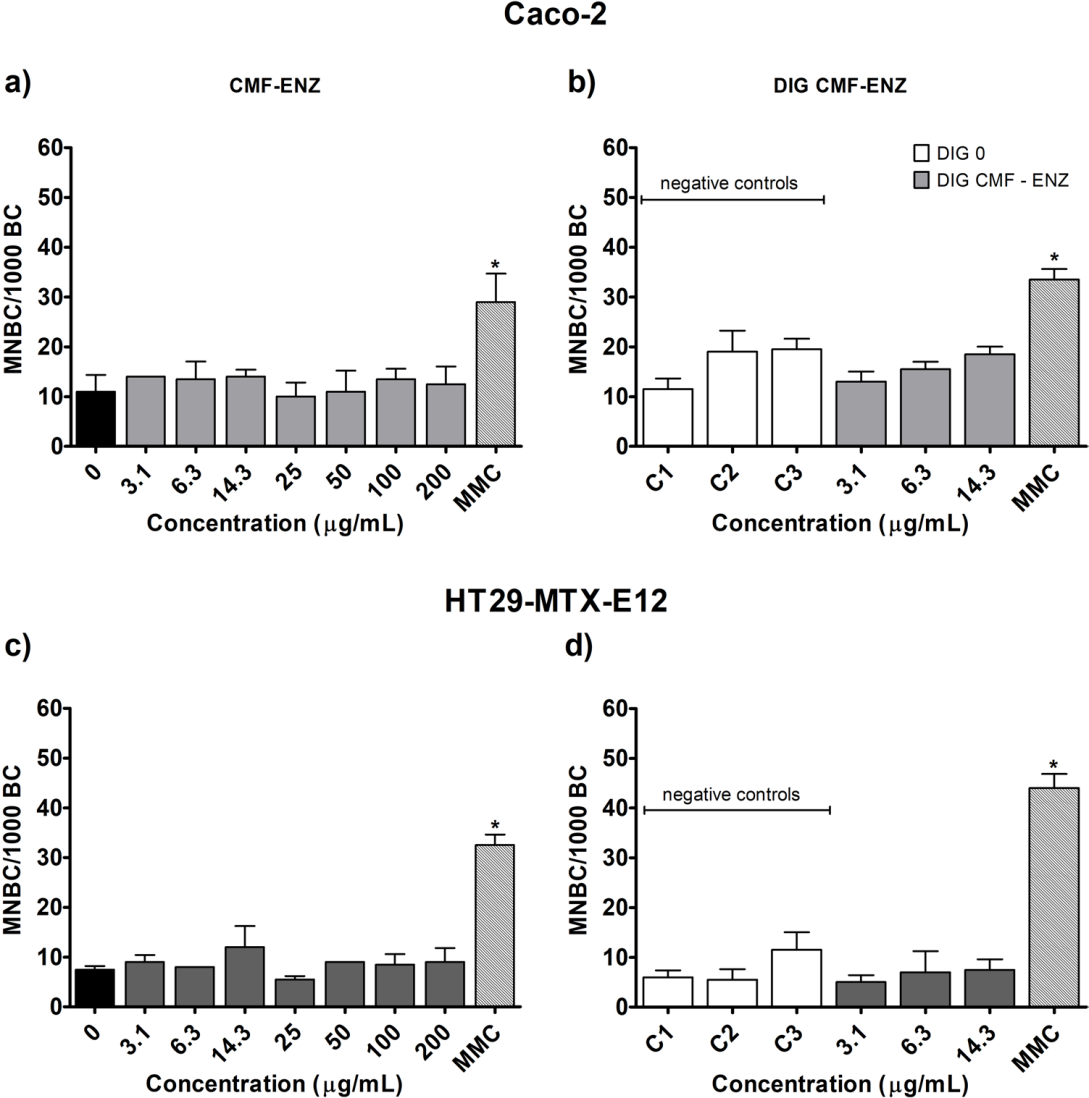
Frequency of micronucleated binucleated cells (MNBC) per 1000 binucleated cells in Caco-2 and HT29-MTX-E12 cells, after exposure to the undigested and digested CMF–ENZ. 0—negative control; C1–C3—DIG 0 controls. Results are presented as mean MNBC/1000 BC ± SD (N = 2). * Significantly different from the respective negative control. Positive control MMC (Mitomycin C: 0.3 μg/mL, 28 h)

**Fig. 8:**
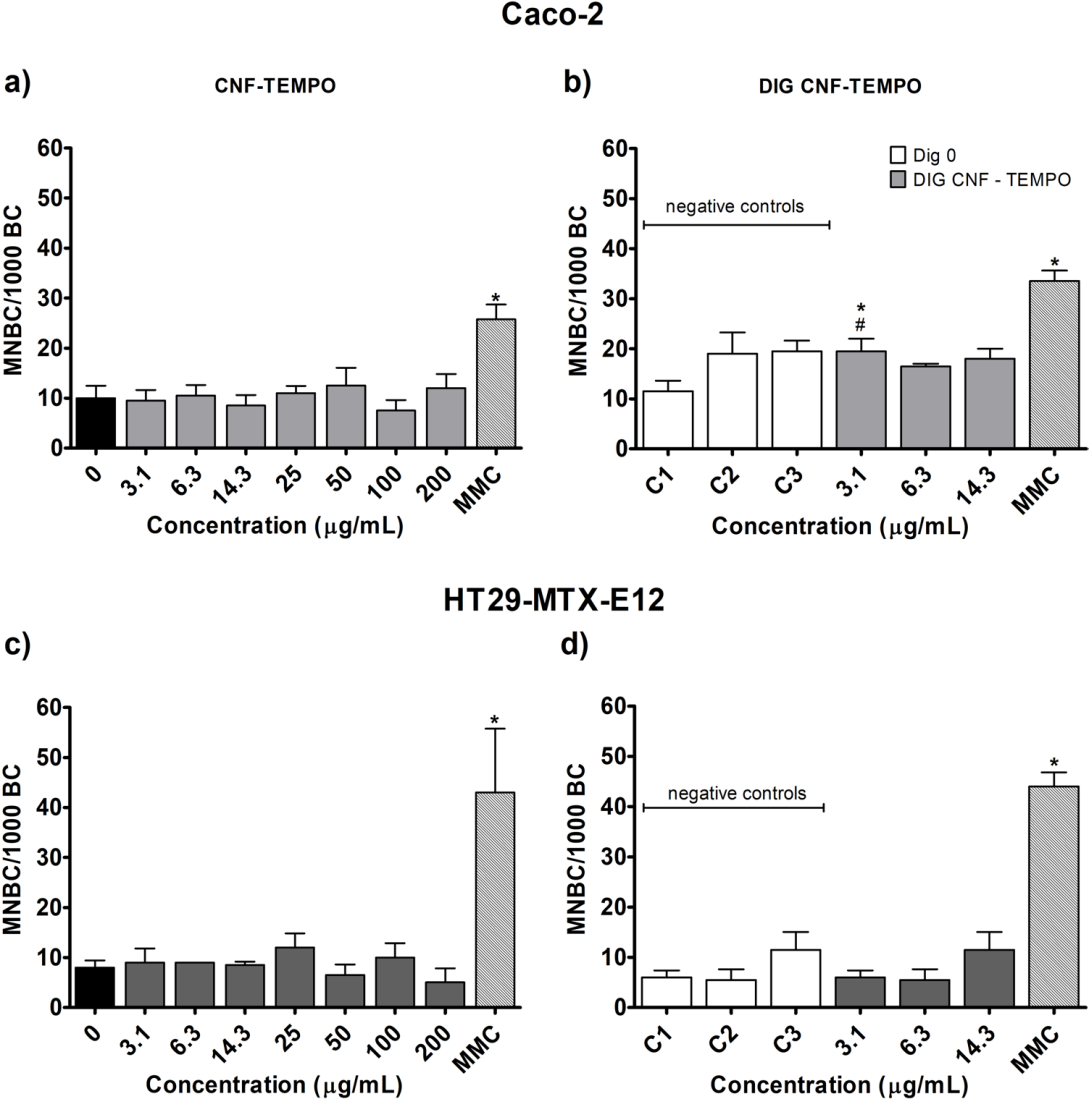
Frequency of micronucleated binucleated cells (MNBC) per 1000 binucleated cells in Caco-2 and HT29-MTX-E12 cells, after exposure to the undigested and digested CNF–TEMPO. 0—negative control; C1–C3—DIG 0 controls. Results are presented as mean MNBC/1000 BC ± SD (N = 2). * Significantly different from the respective negative control. #—Significantly different from the undigested sample. Positive control MMC (Mitomycin C: 0.3 μg/mL, 28 h)

The results of the CBMN assay in Caco-2 and HT29-MTX-E12 cells exposed for 52 h to CMF–ENZ and CNF–TEMPO are presented in Figs. 7 and 8, respectively.

With both cell models, no chromosomal damage was induced with undigested (up to 200 μg/ml) (Fig. 7a, c) or digested CMF–ENZ (up to 14.3 μg/ml) (Fig. 7b, d), when compared to the respective negative controls. The comparison between digested and undigested CMF–ENZ samples at the same concentration did not reveal any differences in the frequency of MNCB/1000 BC (Fig. 7a vs b and Fig. 7c vs d).

Concerning the CNF–TEMPO, no effect was observed after treatment with undigested samples when compared to the respective negative control, irrespectively of the cell line (Fig. 8). However, exposure to 3.1 μg/mL of DIG CNF–TEMPO led to a mild but significant increase in the frequency of MNBC/1000 BC in Caco-2 cells, as compared to DIG 0 (Fig. 8b; C1 vs. 3.1 μg/mL; *p* = 0.0424). In addition, the comparison of results obtained for the same concentrations of digested and undigested CNF–TEMPO showed significant differences, with the MNBC frequency being higher for Caco-2 cells exposure to the concentrations of 3.1 and 14.3 μg/mL of the digested sample (Fig. 8a vs b; *p* = 0.0088, *p* = 0.0128, respectively). However, the increase observed for the highest concentration is biased due to the background effect of the digestion product itself.

Globally, it is concluded that neither the undigested (up to 200 µg/mL) nor the digested CNMs (up to 14.3 µg/mL) induced clastogenic or aneugenic effects in intestinal cells, except in the case of a positive finding in Caco-2 cells, only at the lowest concentration of digested CNF–TEMPO.

### Gene mutation

The concentration range tested in V79 cells was limited by the cytotoxic effects observed in these cells (see Section "CNM’s preparation and concentration range selection"). Regarding the relative platting efficiency (RPE) within the selected dose range, V79 cells did not evidence significant differences between CNMs, digested or not, relatively to the respective negative controls (Fig. S4).

**Fig. 9:**
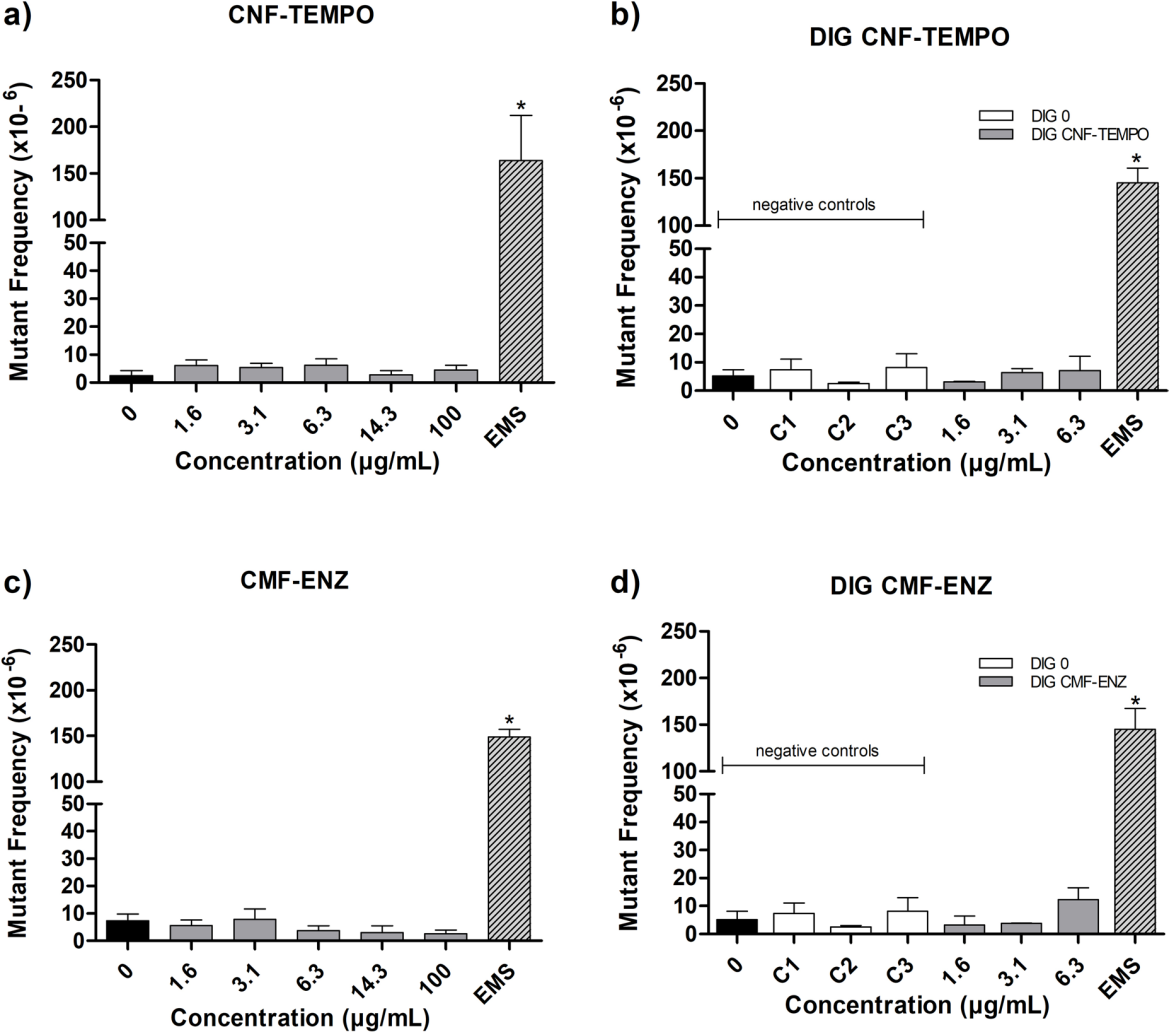
Mutant frequencies in the *Hprt* gene in V79 cells after 24 h of exposure to **a** CNF–TEMPO; **b** digested CNF–TEMPO; **c** CMF–ENZ and **d** digested CMF–ENZ. Data are expressed as the mutant frequency, MF (× 10^−6^) ± SEM (M ± SEM) of two or three independent replicates with two independent harvests pooled together. 0—negative control; C1–C3—DIG 0 controls. *Significantly different from the respective negative control. Positive control EMS (Ethyl Methanesulfonate: 3 mg/mL, 30 min)

With respect to *Hprt* mutant frequency (MF) in V79 cells, after exposure to CNF–TEMPO and CMF–ENZ (Fig. 9), it was not significantly altered after exposure to undigested CNMs, up to 100 μg/mL (Fig. 9a, c) or to digested samples up to 6.3 μg/mL (Fig. 9b,d), when compared to the negative controls. The comparison between digested and undigested samples at the same concentration did not reveal any differences in MF for CNF–TEMPO (Fig. 9a vs b) or for CMF–ENZ (Fig. 9c vs d). The positive control, namely EMS, induced a 28.4-fold increase in the MF relative to the negative control (*p* < 0.0001, Student’s *t* test) in the same cells.

These results show that neither of the CNMs, either digested or non-digested, caused induction of gene mutations in vitro, under the conditions tested.

### Oxidative stress: induction of reactive oxygen species by two CNMs

The intracellular production of ROS after exposure of Caco-2 and HT29-MTX-E12 cells to the different undigested and digested CNMs was determined and is presented, as fold-change relative to the respective control culture medium (0 and DIG 0), in Figs. S5 and S6. Both cell lines did not present any significant increase in ROS, after 3 h or 24 h of exposure to the undigested samples and digested samples, when compared with the respective controls.

### Summary of results

An overview of the results is provided in Table 2. The criteria developed under the Nanogenotox project (NanoGenoTox 2023), together with guidance from OECD on genotoxicity (OECD 2017) were used for the interpretation of the outcome of the assays.

**Table 2:** Summary of outcomes of the toxicological testing of the NMs in this study

	CMF–ENZ				CNF–TEMPO			
	Undigested CNMs		Digested CNMs		Undigested CNMs		Digested CNMs	
	Caco-2	HT29-MTX-E12	Caco-2	HT29-MTX-E12	Caco-2	HT29-MTX-E12	Caco-2	HT29-MTX-E12
Cytotoxicity assays								
MTT Assay (24 h Exposure)	–	–	–	–	−	−	−	−
Clonogenic (9 day exposure, HT29-MTX-E12; 15 day exposure Caco-2)	–	–	a)	a)	−	−	a)	a)
Replication Index (RI) (52 h Exposure)	–	–	–	–	−	−	−	−
Cytokinesis-Block Proliferation Index (CBPI) (52 h Exposure)	–	–	–	–	−	−	−	−
Oxidative stress								
ROS (3 h Exposure)	–	–	–	–	−	−	−	−
ROS (24 h Exposure)	–	–	–	–	−	−	−	−
Genotoxicity assays								
Conventional alkaline Comet Assay (3 h Exposure)	(+)	+	–	(+)	+	+	(+)	(+)
Conventional alkaline Comet Assay (24 h Exposure)	+	(+)	–	–	(+)	−	(+)	−
FPG-modified Comet Assay (3 h Exposure)	+	–	–	–	(+)	(+)	−	−
FPG-modified Comet Assay (24 h Exposure)	(+)	+	(+)	–	+	(+)	−	−
Cytokinesis-Block Micronucleus Assay (CBMN) (52 h Exposure)	–	–	–	–	−	−	(+)	−
Gene mutation in V79 cells (24 h exposure)	–		–		–		–	

+++, POSITIVE—Significant concentration−dependent increase, ≥2 significant concentrations; ++, POSITIVE—Significant concentration−dependent increase, high concentration significant; +, POSITIVE—No significant concentration−dependent increase, ≥2 significant concentrations; (+), EQUIVOCAL—No significant concentration−dependent increase, 1 significant dose; −, NEGATIVE—none of the criteria for a positive result are met. CBPI—cytokinesis−block proliferation index, RI—replication Index, CBMN—cytokinesis−block micronucleus assay, FPG—formamidopyrimidine DNA glycosylase. ROS—reactive oxygen species. a) clonogenic assay resulted in cell death due to toxicity of the digestion product itself

## Discussion

In the present study, we assessed the safety of two types of nanocelluloses that are under development for possible application in food-related products. This study was set as an early stage screening strategy for toxicity during the development of these materials, as proposed in the framework of a safe-and-sustainable by design approach (SSbD). Genotoxicity endpoints of major regulatory relevance were addressed using an integrated in vitro approach including assays for aneugenicity/clastogenicity, DNA damage, and gene mutation, performed according to current guidelines and adding also a new approach methodology for in vitro human digestion, prior to genotoxicity tests.

The present work shows that neither CNF–TEMPO nor CMF–ENZ are cytotoxic, in Caco-2 or HT29-MTX-E12 intestinal cells, up to 200 µg/mL, after 24 h of exposure, i.e., did not reduce cell viability by more than 30%, as defined by the ISO standard 10993–5 (ISO 2009). For both CNMs tested, no effects on cells division were detected after 52h exposure, and the ability of cells to divide and form colonies was not affected even after a longer exposure period, i.e., 9 and 15 days. These data are suggestive of the biocompatibility of CNMs in intestinal cells. Accordingly, in Caco-2 cells, most studies indicated no cytotoxicity, when using other CNMs up to 2000 µg/mL, independently of the source of the material and production method (Coelho et al. 2018; Tibolla et al. 2018, 2019; González-Domínguez et al. 2019; Xiao et al. 2019; Chen et al. 2020; Lopes et al. 2020; Vincentini et al. 2023). Negative results were also reported for other intestinal cells (HCT116, HT-29, and CCD112 colon fibroblast 2D cells) treated with concentrations below 500 µg/mL (Hanif et al. 2014; Vakili et al. 2021; Yusefi et al. 2022). However, one study reported cytotoxic effects of three CNMs after exposure of differentiated Caco-2 cells to 50 μg/mL, while other five CNMs were negative, including a CNF produced by TEMPO-mediated pre-treatment (Mortensen et al. 2022). The data from the later study suggested that the diameter and length of CNMs have an important role in the interaction between CNMs and enterocytes, whereas the level of aggregation in cell medium had no impact on the biological effect. Conversely, a few positive findings reported corresponded to concentrations above 2000 µg/mL (Tibolla et al. 2018, 2019). The cytotoxicity observed at very high concentrations has been attributed to the tendency to gel formation which may hamper the gas exchange through the cell membranes (Hanif et al. 2014). CNMs with specific functionalization, such as anionic CNFs produced by carboxymethylation induced cytotoxic effects (Lopes et al. 2020). Moreover, the amounts of carboxyl groups on the surface of the CNMs may influence its toxic effects. A charge-dependent decrease in Caco-2 mitochondrial activity was reported only for cellulose nanocrystals with a carboxyl content higher than 3.8 mmol/g after 24h exposure to CNCs (50–300 µg/mL) with various amounts of surface carboxyl groups (Hosseinidoust et al. 2015). No cytotoxic effects were observed after Caco-2 exposure to four carboxymethylated CNFs (100–1000 µg/mL) with carboxyl contents below 3.8 mmol/g, for 24 h (Zhang et al. 2021). In our study, the carboxyl content of CNF–TEMPO (1.33 mmol/g) and CMF–ENZ (0.143 mmol/g) was considerably below the reported threshold. In more complex intestinal co-culture models, the biocompatibility of the CNMs was suggested by the absence of cytotoxic effects following direct exposure to CNFs or CNCs (0.4% w/w) for 1–48 h and to digested samples after using the INFOGEST protocol (Ede et al. 2020; Pradhan et al. 2020). In those reports, the low concentrations of digestion product used allowed the biological testing after simulated digestion without important effects on toxicity. After 24h exposure of co-culture models to CNCs, CNFs and FITC-tagged CNFs, at concentrations up to 1.5% w/w, using a different in vitro static digestion protocol, no cytotoxicity was observed (Deloid et al. 2019; Salari et al. 2019; Patel et al. 2021). A potential protective effect of the digestion process has been reported when assessing in Caco-2 the cytotoxic effect of another carbon-based NM, graphene oxide, by application of a different in vitro digestion protocol (Cebadero-Domínguez et al. 2023).

The positive results in the comet assay indicated that both CNMs induce DNA damage (single and double DNA strand breaks and alkali-labile sites) in Caco-2 and HT29-MTX-E12 intestinal cells. Despite the low levels of DNA damage observed, they were above the levels of the negative controls and revealed a genotoxic potential of the CNMs under study. It must be noted that the positive effects are not so evident after the in vitro digestion, since in that situation the DNA damaging potential of CNMs was considered equivocal. In the literature, only one study reported DNA-damaging effects of CNMs in intestinal cells with the comet assay, following exposure to 500 µg/mL of cationic CNCs (Mahmoud et al. 2023). However, the high concentration tested limited the relevance of the result. No effects were reported in Caco-2 cells exposed for 24h to a set of CNMs (0.4–120 μg/mL), using other DNA damage markers for DNA double-strand breaks, γ-H2AX and the ATM phosphorylation analysis (Vincentini et al. 2023).

Although our study raises concerns about the genotoxicity of these CNM in human intestinal cells, the comet assay is considered as an indicator test that can provide complementary information to endpoints such as gene mutation and chromosomal damage (EFSA Scientific Committee et al. 2021). The DNA damage observed in the comet assay might be due to a direct interaction of the DNA molecule and the nanocellulose fibres that cross the membrane barrier and nuclear membrane, reaching the nucleus. In addition, oxidative stress has been considered as a possible indirect mechanism underlying NMs’ toxicity/genotoxicity, but the present data do not evidence neither induction of ROS, directly or after the digestion process nor a relevant oxidant effect on DNA (FPG-modified comet assay). The absence of ROS generation might have been due to the timepoint selected (3h and 24h), since ROS induction is transient and rapidly eliminated by cells. However, its DNA damaging effect is generally captured at 3h post-exposure by using the modified version of the comet assay. Our negative results are in line with the absence of ROS generation after 24 h exposure of Caco-2 cells to a panel of CNFs (0.4 to 120 μg/mL) (Vincentini et al. 2023) or in tri-cultures systems, after in vitro digestion and different exposure times to CNCs and CNFs (Ede et al. 2020; Pradhan et al. 2020). Deloid et al. (2019) reported the induction of ROS only when tri-cultures where exposed to 1.5% w/w CNC (Deloid et al. 2019). Furthermore, in respiratory cells, negative results were also described for BEAS-2B exposed for 3–24 h to CNF without any functionalization or to CNF produced by TEMPO-mediated oxidation up to concentrations of 250 μg/mL (Aimonen et al. 2022). In a previous study, no ROS induction occured in A549 cells after 1 h and 24 h exposure to the same CNF–TEMPO and CMF–ENZ samples used in this study (Pinto et al. 2022). However, low concentrations (1.5 and 3 μg/cm^2^) of CMF–ENZ were able to induce DNA oxidation lesions in co-cultures of A549 and THP-1 cells, possibly associated with the immune response of the macrophage-like (THP-1) cells (Ventura et al. 2023). Considering the “fibre-like” morphology of CNMs, it is plausible that an inflammatory response occurs, like for some carbonaceous fibres and/or asbestos (Ventura et al. 2018; 2020; Pinto et al. 2022;). Further mechanistic understanding of the effects observed is expected from underway whole genome methylation analysis regarding these exposed intestinal cells.

In the present work, the in vitro micronucleus assay did not cause chromosomal damage in intestinal cells exposed to either CNMs, directly or after digestion simulation. To the best of our knowledge, no studies have investigated the effects of CNMs on chromosome stability in intestinal cells. Different outcomes have been reported in non-intestinal cell lines, depending on the type of CNMs and cell models used. The same CNF–TEMPO used in the present study and also one CNC did not induce micronuclei formation in A549 cells (Pinto et al. 2022). There was, however, a chromosomal damaging effect, at the lowest (1.5 µg/cm^2^) and the highest (50 µg/cm^2^) concentrations of CMF–ENZ in exposed A549 cells (Pinto et al. 2022). In addition, exposure to 4.8 µg/mL of CMF–ENZ increased the MN frequency in MG-63 cells, but not in V79 cells (Ventura et al. 2022) and no genotoxicity was observed for that sample in co-cultures of A549 and THP-1 cells, using the micronucleus assay (Ventura et al. 2023). Interestingly, using a different CNF–TEMPO sample, with a lower carboxyl group content (1177 µmol/g), and a higher degree of polymerization and fibre diameter (18.5 nm), positive results had been previously reported in the MN and comet assays, at a low concentration range (4.8–9.6 µg/mL) in A549 and THP1 co-cultures (Ventura et al. 2018). In addition, exposure to 40 µg/mL of that same CNF–TEMPO had increased MN frequency in V79 cells (Ventura et al. 2022).

Studies by other authors have also generated somewhat inconsistent data on the capacity of nanocelluloses to induce chromosomal instability. In human bronchial epithelial BEAS-2B cells, overall neither induction of micronucleus nor DNA damage was reported after exposure to CNFs from different sources and methods, including by enzymatic pre-treatment (Lindberg et al. 2017; Aimonen et al. 2021). In another study with BEAS-2B, in which the different types of CNFs were size fractionated into fine, medium, and coarse fractions, chromosomal and DNA damage was observed only after exposure to the highest concentrations (333 and 1000 µg/ml) of the fine fraction of the cationic CNF functionalized with epoxypropyltrimethylammonium chloride (Aimonen et al. 2022). Increased MN frequency was also observed after exposure to a coarse fraction of carboxymethylated CNF (Aimonen et al. 2022). Negative results were reported in the chromosomal aberration test (OECD-compliant), in Chinese hamster lung fibroblasts (CHL/IU), after 6h exposure to 25–100 μg/mL of CNFs, that were produced either via TEMPO oxidation or via mechanical defibrillation of needle-type bleached kraft pulp (Fujita et al. 2021). These and our results support the widespread perspective related to the toxicity assessment of nanomaterials, stating that changes in the physicochemical properties of closely related nanomaterials can affect their toxicity/genotoxicity.

It is noted that, after using the in vitro digestion, CNF–TEMPO was considered to produce equivocal results on chromosomal damage in Caco-2 cells, in view of positive findings after exposure to one concentration. Considering the low concentrations that could be tested, due to the cytotoxicity of the digestion product, this result raises concerns on the ability of this CNM causing chromosomal damage.

In the mammalian gene mutation assay, there was no increase in the MF either after exposure to CNF–TEMPO or CMF–ENZ, undigested or after simulated digestion. In addition, the digestion process did not increase the MF for either CNMs, showing no differences between the digested and undigested samples. In spite pulmonary cells were used for the evaluation of gene mutation, these are considered as representative of any potential mutagenic effect occurring in mammalian cells (OECD 2016). Currently, no further studies regarding gene mutation after exposure to CNMs have been reported using the HPRT test. Gene mutation was evaluated in one study using the mouse lymphoma TK assay (cell line L5178Y tk ± 3.7.2C) with two CNFs, one produced by TEMPO-oxidation and other produced via mechanical defibrillation of needle bleached kraft pulp, showing no induced gene mutations up to 100 μg/mL (Fujita et al. 2021). Conversely, to our knowledge, the in vitro digestion of CNMs was never applied together with gene mutation assays.

Overall, this work shows that the studied CNMs did not decrease cellular viability and proliferation at the tested concentrations up to 200 µg/mL in intestinal cells. The comet assay indicated that both CNMs induced in vitro DNA damage, despite the very low levels of damage observed. Yet, no dose–response was observed. While the three criteria for a clear positive result (OECD 2017) are not met in the comet assay, none of the CNMs can be considered clearly negative. Applying the Nanogenotox criteria, both CMF–ENZ and CNF–TEMPO are considered positive in the two cell lines, due to positive findings at least in one sampling time. No increase in ROS levels attributable to CNMs was observed, under the tested conditions. The two CNMs did not induce chromosomal damage in the two intestinal cells.

The relevance of using the in vitro digestion in genotoxicity assessments is highlighted in the current work as a new approach methodology to advance toxicological studies, to better mimic the effect of these NMs in the GIT, moving towards non-animal methods. However, the digestion product, which include digestive enzymes and bile salts, was shown to have an impact on intestinal cells’ death, thus creating challenges for its downstream use in toxicological assays, particularly when a food matrix has not been considered (Vital et al. 2024). In the present results, the digestion process slightly impacted the genotoxic effects observed with the CNMs (decreased the level of DNA damage and increase chromosomal damage of one CNF–TEMPO concentration), when considering the concentration range that was possible to test for digested CNMs for each endpoint.

Although CNMs are generally advertised in industrial and scientific communities as nontoxic and biocompatible, the current body of knowledge is still insufficient to guarantee CNMs safe use, particularly through ingestion. By using an integrated approach including a test battery of in vitro assays we show that two different nanocelluloses produced from industrial bleached *Eucalyptus globulus* kraft pulp by different pre-treatments, CNF–TEMPO and CMF–ENZ, do not induce gene mutations or aneugenic/clastogenic chromosomal damage i*n vitro*, with promising results towards potential applications in food technology. However, due to the indication of DNA damage induction, the genotoxic potential of the examined CNMs cannot be totally excluded. The in vitro human digestion seems to attenuate the effects of increased DNA damage. On the contrary, the lowest concentration of digested CNF–TEMPO induced chromosomal damage in Caco-2 cells, leading to an equivocal outcome. Ongoing research on epigenotoxic effects of these CNMs samples or future research using in vivo models may strengthen the lines of evidence on their safety when ingested, paving the way for their innovative application in the food industry.

## Supplementary Information

Below is the link to the electronic supplementary material.

Supplementary file1 (PDF 2140 KB)

## Data Availability

The data that support the findings of this study are available from the corresponding author, upon request.
