# Supplementary material for: Evaluation of the cyto- and genotoxicity of two types of cellulose nanomaterials using human intestinal cells and in vitro digestion simulation
Source: Arch Toxicol. 2024 Dec 24;99(2):575–96. doi: 10.1007/s00204-024-03911-2 (PMC11775080; doi:10.1007/s00204-024-03911-2)
Supplement: Supplementary file 1 — Supplementary file1 (PDF 2140 KB) [file 204_2024_3911_MOESM1_ESM.pdf]

Supplementary information

Evaluation of the cyto- and genotoxicity of two types of cellulose nanomaterials using human intestinal cells and *in vitro* digestion simulation

Nádia Vital[1,2,3], Maria Cardoso[1], Michel Kranendonk [2,3], Maria João Silva [1,3]*, and Henriqueta Louro[1,3]

[1] National Institute of Health Dr. Ricardo Jorge, Department of Human Genetics, 1649-016 Lisbon, Portugal

[2] NOVA Medical School, Universidade NOVA de Lisboa, 1169-056 Lisbon, Portugal

[3] Centre for Toxicogenomics and Human Health (ToxOmics), NOVA Medical School, Universidade NOVA de Lisboa, 1169-056 Lisbon, Portugal

* Corresponding author:

Maria João Silva

Department of Human Genetics

Research and Development Unit

National Institute of Health Doutor Ricardo Jorge

Avenida Padre Cruz, 1649-016 Lisboa, PORTUGAL

TEL +351 217 519 234

m.joao.silva@insa.min-saude.pt

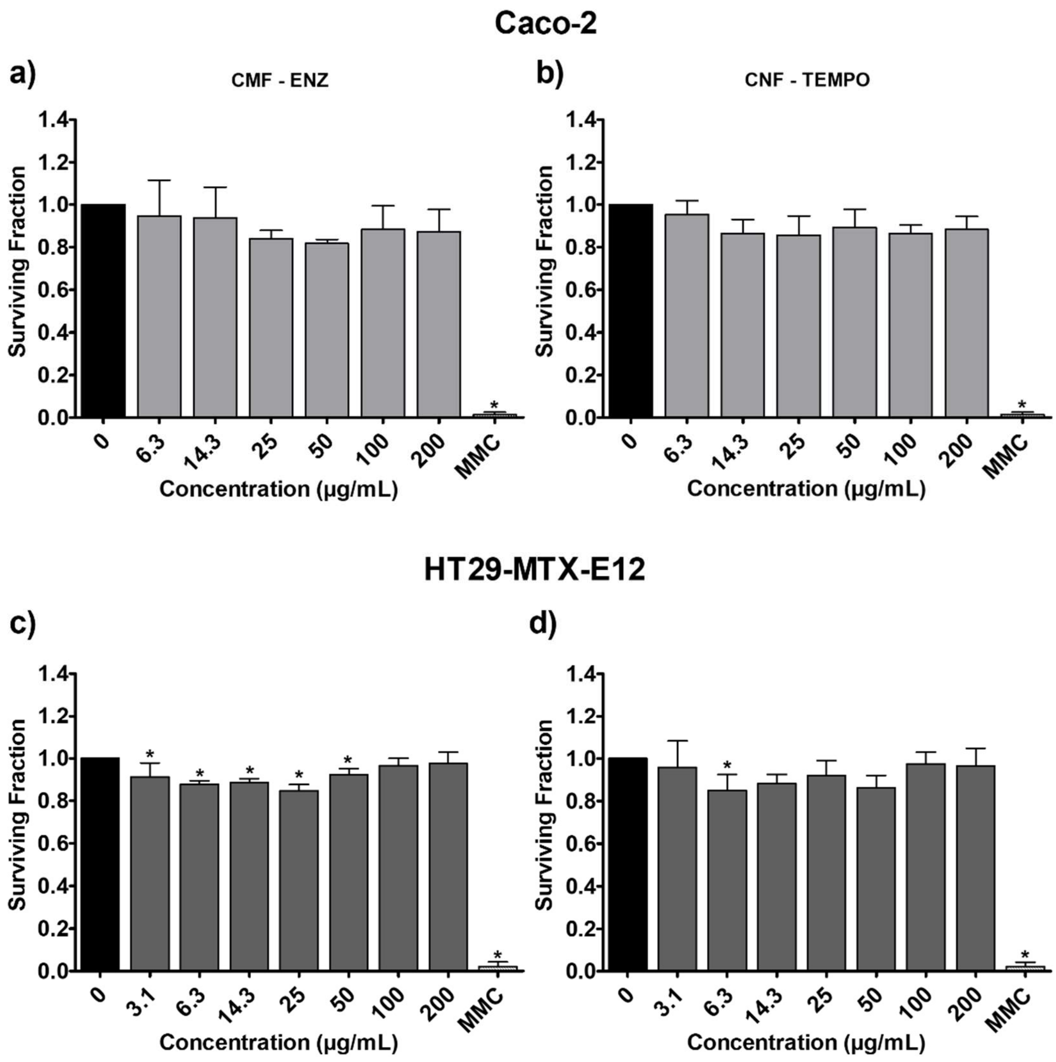

Fig. S1 Cell survival (clonogenic assay) of Caco-2 or HT29-MTX-E12 cells when exposed to undigested CMF-ENZ and CNF-TEMPO. Results are presented as mean surviving fraction ± Standard deviation (N = 4). * - Significantly different from the negative control. Positive control MMC (Mitomycin C: 0.05 μg/mL or 0.025 μg/mL, for Caco-2 and HT29-MTX-E12, respectively; 24h).

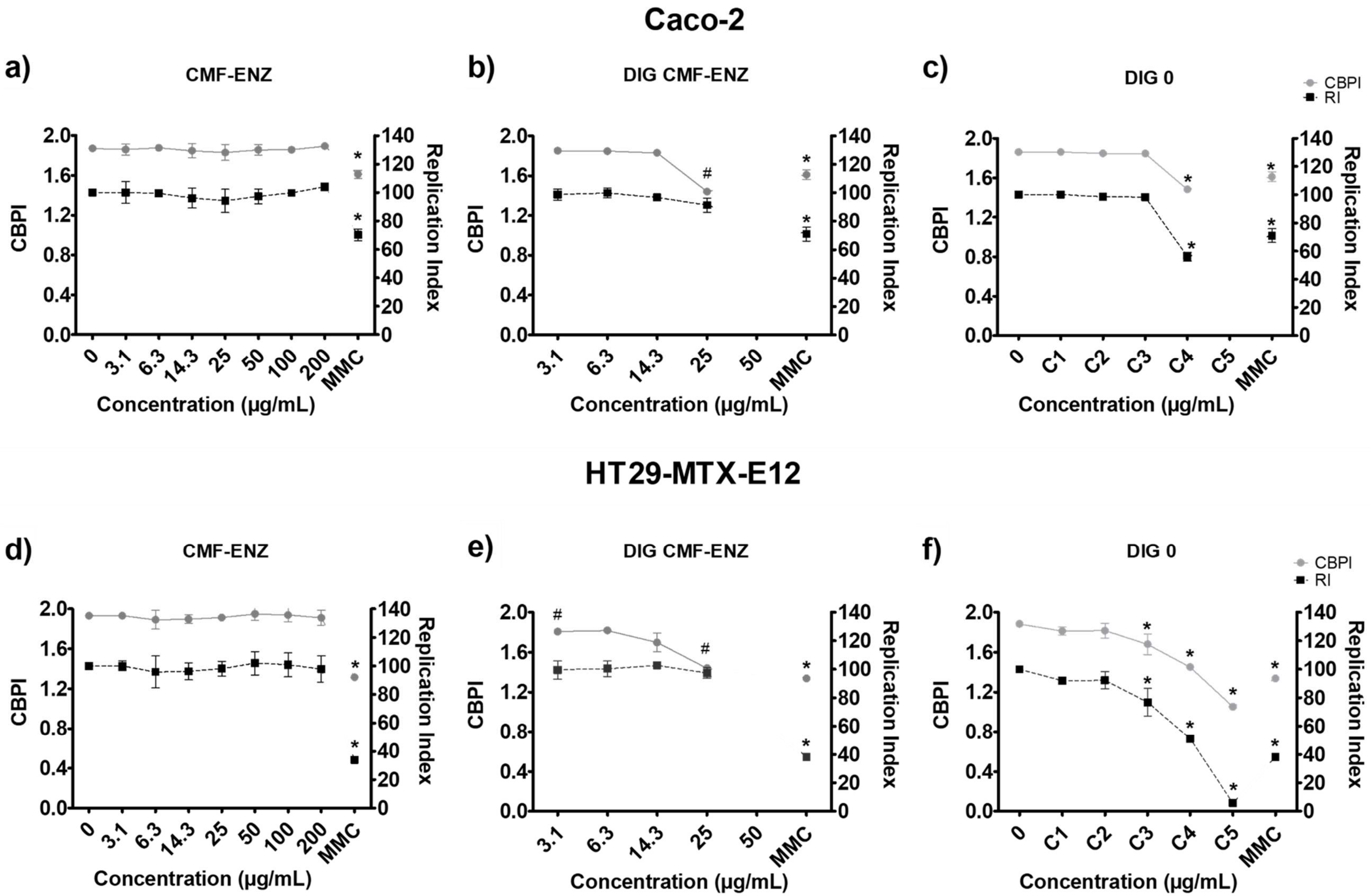
Caco-2
a)
CMF-ENZ
b)
DIG CMF-ENZ
c)
DIG 0
CBPI
RI
CBPI
Replication Index
Concentration (µg/mL)
0
3.1
6.3
14.3
25
50
100
200
MMC
C1
C2
C3
C4
C5
HT29-MTX-E12
d)
CMF-ENZ
e)
DIG CMF-ENZ
f)
DIG 0

Fig. S2 CBPI and RI of Caco-2 and HT29-MTX-E12 cells, after 52 h exposure to undigested and digested CMF-ENZ. Graphics **c** and **f**. show the results from CBPI and RI after exposure only to digestion product controls (DIG 0), for each cell line. Results are presented as mean ± Standard deviation (N = 2). 0 - negative control; * Significantly different from the respective negative control. **#** - significantly different from undigested CNMs. Positive control MMC (Mitomycin C: 0.3 µg/mL, 28 h).

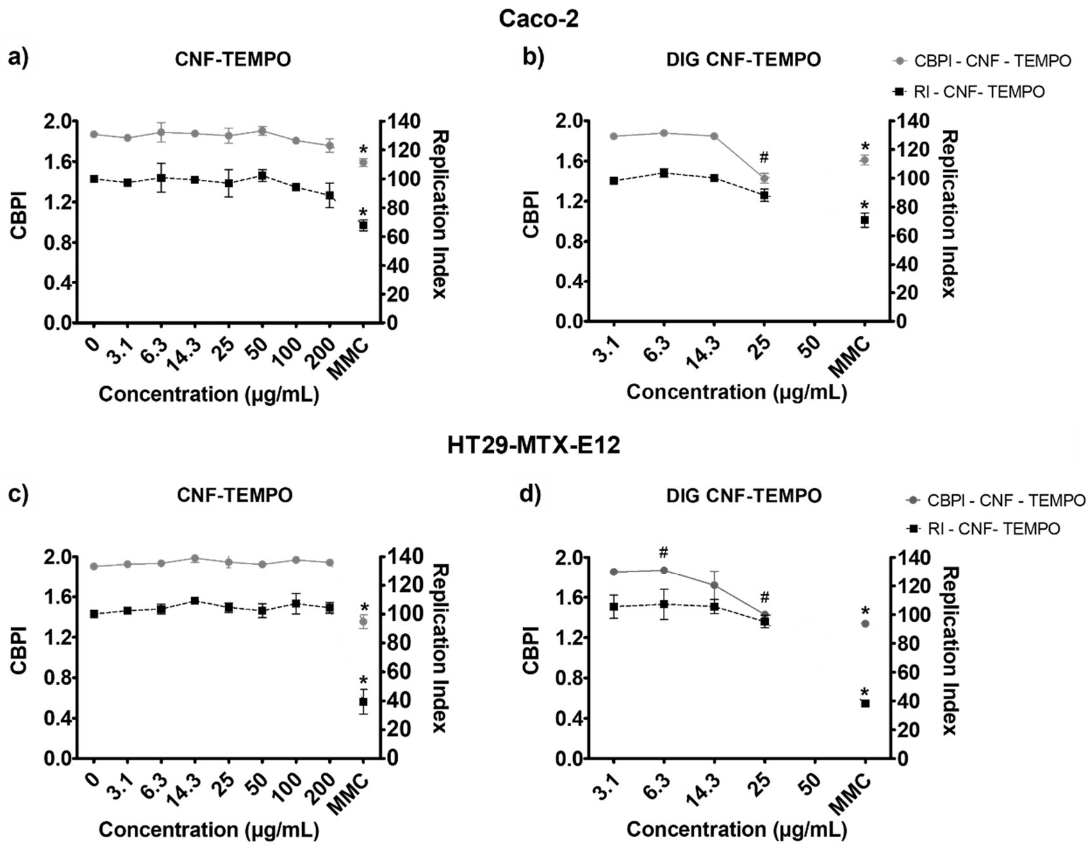

Fig. S3 CBPI and RI of Caco-2 and HT29-MTX-E12 cells, after 52 h exposure to undigested and digested CNF-TEMPO. Results are presented as mean ± Standard deviation (N = 2). 0 - negative control; * Significantly different from the respective negative control. **#** - significantly different from undigested CNMs. Positive control MMC (Mitomycin C: 0.3 µg/mL, 28 h).

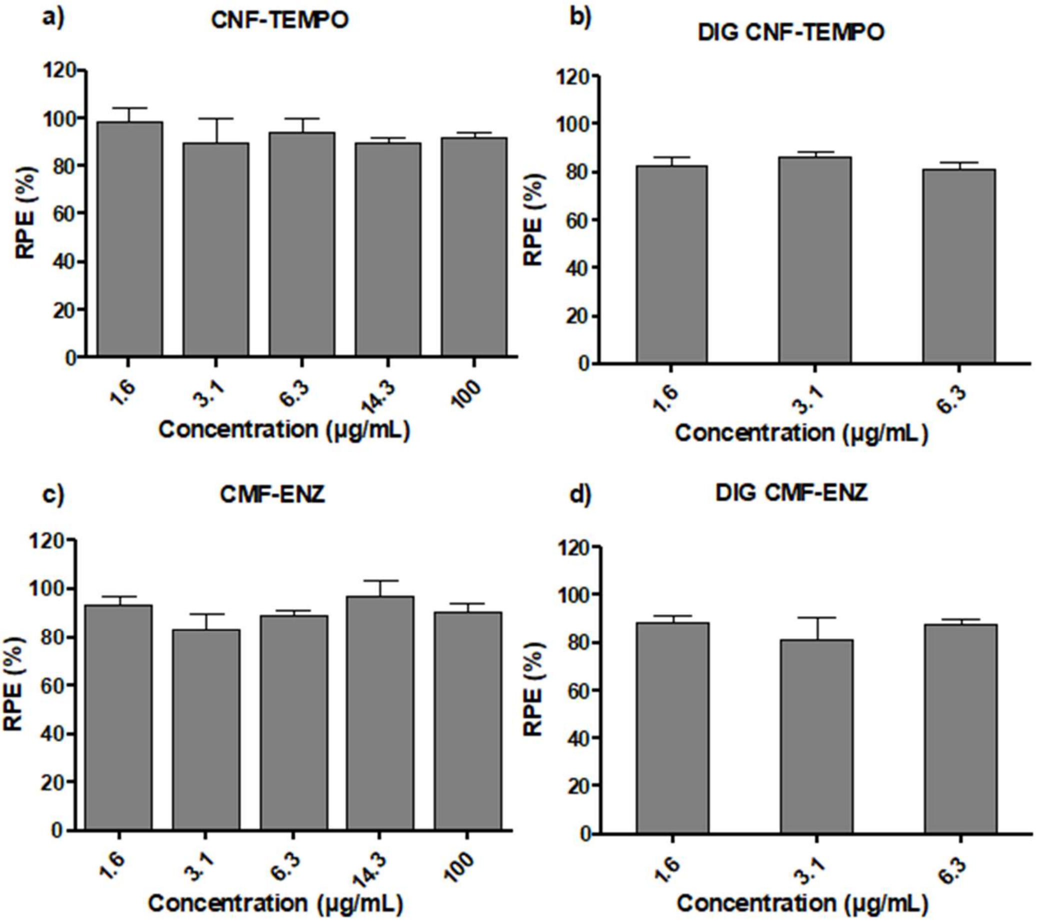

Fig. S4 Cytotoxicity effect measured by Plating efficiency (PE) assay after 24 hours exposure of V79 cells to a) CNF-TEMPO; b) Digested CNF-TEMPO; c) CMF-ENZ and d) Digested CMF-ENZ. Bars represent cytotoxicity relative to 100 % of untreated cells, expressed as mean ± SEM of two or three independent experiments. 0 - negative control; C1-C3 - DIG 0 controls.

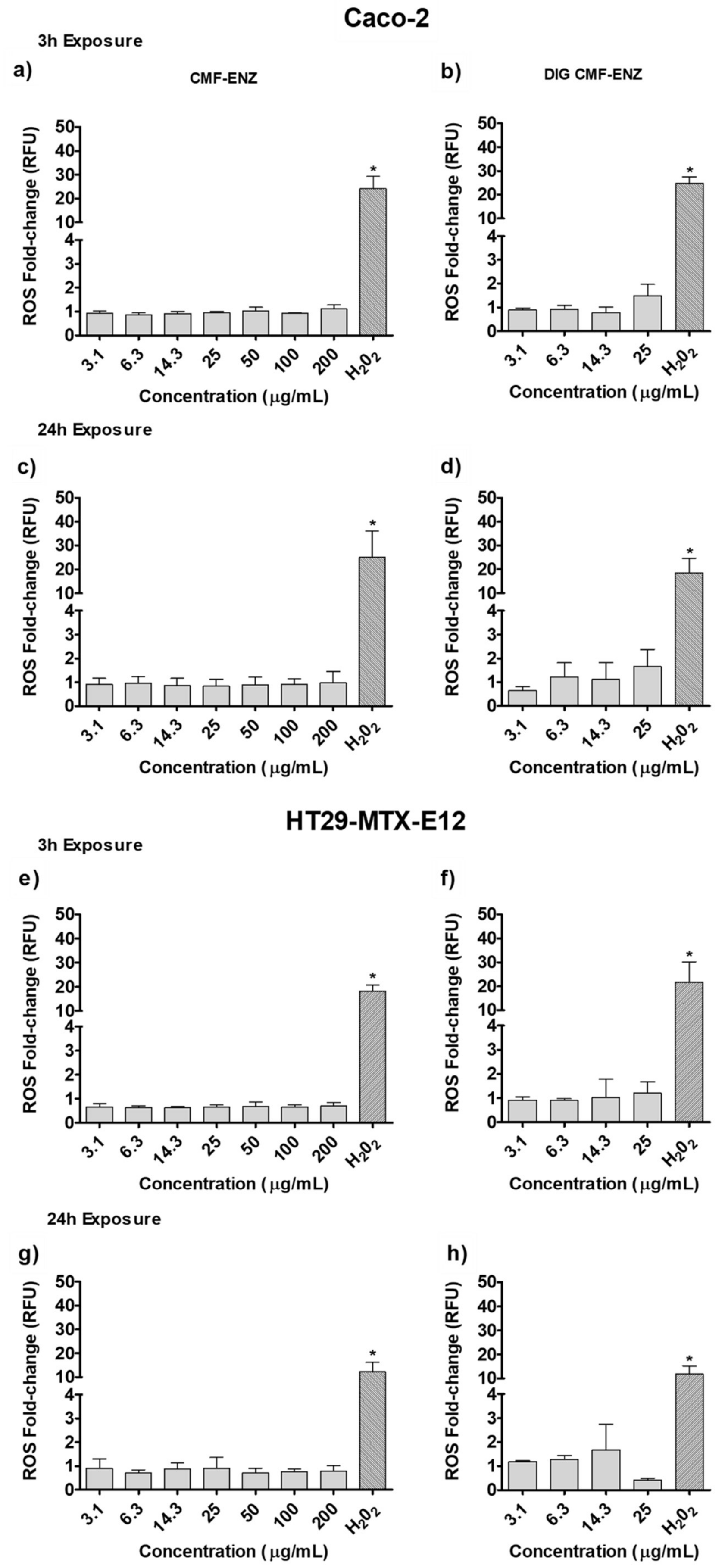
Caco-2
3h Exposure
a)
CMF-ENZ
b)
DIG CMF-ENZ
24h Exposure
c)
d)
HT29-MTX-E12
3h Exposure
e)
f)
24h Exposure
g)
h)
ROS Fold-change (RFU)
0
1
2
3
4
10
20
30
40
50
3.1
6.3
14.3
25
50
100
200
H_2O_2
*
Concentration (μg/mL)

Fig. S5 Intracellular ROS levels (DCFDA assay) in Caco-2 and HT29-MTX-E12 cells after 3 h and 24 h exposure to undigested and digested CMF-ENZ. Results are presented as relative fluorescence units (RFU) compared to the respective control cells (mean ± Standard deviation; N = 3). Positive control H_2O_2 (250 µM, 1 h).

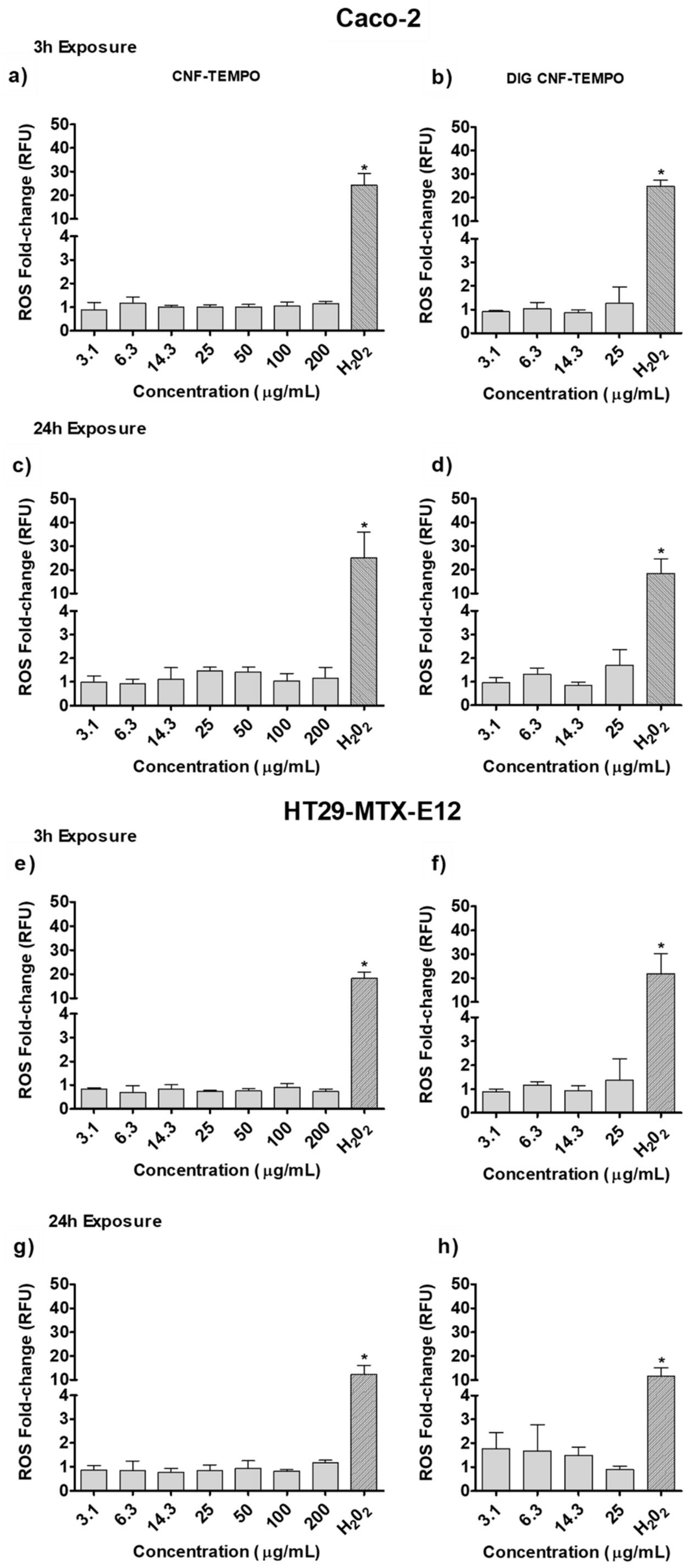
Caco-2
3h Exposure
a)
CNF-TEMPO
b)
DIG CNF-TEMPO
24h Exposure
c)
d)
HT29-MTX-E12
3h Exposure
e)
f)
24h Exposure
g)
h)
ROS Fold-change (RFU)
Concentration (μg/mL)
3.1
6.3
14.3
25
50
100
200
H2O2
*

Fig. S6 Intracellular ROS levels (DCFDA assay) in Caco-2 and HT29-MTX-E12 cells after 3 h and 24 h exposure to undigested and digested CNF-TEMPO. Results are presented as relative fluorescence units (RFU) compared to the respective control cells (mean ± Standard deviation; N = 3). Positive control H_20_2 (250 µM, 1 h).
